# Controlling the Sulfation Density of Glycosaminoglycan Glycopolymer Mimetics Enables High Antiviral Activity against SARS-CoV‑2 and Reduces Anticoagulant Activity

**DOI:** 10.1021/acs.biomac.5c00576

**Published:** 2025-07-07

**Authors:** Miriam Hoffmann, Lorand Bonda, Ines Fels, Darisuran Anhlan, Eike Hrincius, Derik Hermsen, Stephan Ludwig, Mario Schelhaas, Nicole L. Snyder, Laura Hartmann

**Affiliations:** † Department of Organic and Macromolecular Chemistry, 9170 Heinrich-Heine-University Düsseldorf, Universitätsstraße 1, Düsseldorf 40225, Germany; ‡ Institute of Cellular Virology, ZMBE and Cells in Motion Interfaculty Centre CiMIC, 9185 University of Münster, Münster 48149, Germany; § Institute of Molecular Virology, ZMBE, University of Münster, Münster 48149, Germany; ∥ Central Institute of Laboratory Medicine, Medical Faculty, University Hospital Düsseldorf, Heinrich-Heine-University, Düsseldorf 40225, Germany; ⊥ Department of Chemistry, 2813 Davidson College, Davidson, North Carolina 28035, United States; # Institute for Macromolecular Chemistry, 9174 University of Freiburg, Stefan-Meier-Street 31, Freiburg i.Br. 79104, Germany

## Abstract

Sulfated glycosaminoglycans (sGAGs) make up a class of cell-surface glycans known to mediate pathogen engagement. Glycopolymers mimicking sGAGs can reduce or prevent pathogen attachment. However, their high anticoagulant activity limits their biomedical applications. Here, we report the synthesis and evaluation of synthetic glycopolymers mimicking sGAGs with high antiviral activity but low anticoagulant activity. The key lies in the control of the density of carbohydrates presented along the polymeric backbone. This was accomplished via copolymerization of carbohydrate with noncarbohydrate monomers. We reveal that the polymer chain length affects inhibition of SARS-CoV-2 pseudovirus (PsV) and authentic virus infections, and that above a critical chain length, density of carbohydrate and sulfate groups can be reduced, maintaining high antiviral activity while minimizing anticoagulant activity. This demonstrates, for the first time, how specific structural parameters of glycopolymers can be used to maximize inhibition while minimizing anticoagulative properties unlocking the full potential of sGAG mimetics in fighting infections.

## Graphical abstract

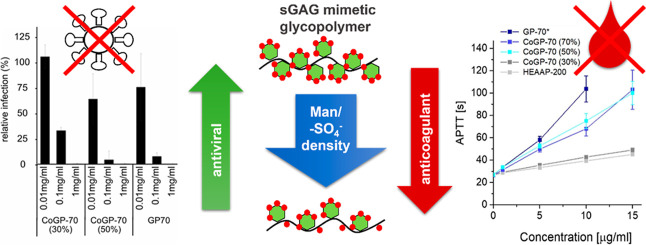

## 1 Introduction

The severe acute respiratory syndrome coronavirus 2 (SARS-CoV-2) continues to threaten human health. Despite the availability of vaccines as the gold standard of protection against severe SARS-CoV-2 infection outcomes, the virus continues to evolve through immune-evading mutations. This has led to an interest in supportive therapeutics with the potential to target mechanisms of the infection process that are less likely to be prone to such fast mutations. One such mechanism is the first step of the infection process, the attachment of the virus to the host cell surface, which is typically followed by cell entry. Developing inhibitors to block this attachment can lead to therapeutics for protection against and treatment of SARS-CoV-2 infection. SARS-CoV-2, like many other viruses, is known to engage proteoglycans on the host cell surface.^1–3^ Proteoglycans are composed of a membrane-anchored protein with long polysaccharide brush-like side chains which serve as attachment factors at the cell surface. One of the primary polysaccharides engaged by SARS-CoV-2 is heparan sulfate (HS).^3–7^ HS is a member of the glycosaminoglycan (GAG) family, which comprises linear and negatively charged polysaccharides that are characterized by a high structural diversity.^8,9^

In recent years, several groups have interrogated the ability of HS and heparin (HP), a more highly sulfated GAG related to HS, to inhibit SARS-CoV-2.^10,11^ These efforts have demonstrated that the application of such compounds can significantly reduce or even completely block infection, which can open up new therapeutic modalities for prophylactic treatments and/or acute infection. These findings have even led to the recommendation of using HP in the treatment against SARS-CoV-2 infection.^12,13^ However, such analogues are also well known for their anticoagulant activity, leading to the risk of undesired off-target effect.^14^ Furthermore, HS/HP, like many other glycosaminoglycans, are notoriously heterogeneous^15–17^ which can make it difficult to understand the specific structure/function relationships that govern their interactions with viruses as well as increase off-target effects. This has inspired the preparation of HS/HP mimetics which allow for better structural control,^18–22^ and in turn, this work has more recently been applied to address SARS-CoV-2.^23–29^ One class of sGAG mimetics applied to SARS-CoV-2 is the sulfated polyglycerols. Recent results by Nie et al. demonstrated that sulfated linear and branched polyglycerols could drive interactions with the virus, primarily through electrostatic interactions.^30^ This is indeed particularly relevant for SARS-CoV-2 as it has been observed that during mutation of the virus, an increase in cationic amino acids in and near the region identified as the primary HS recognition site occurred. A second class of sGAG glycopolymer mimetics not only carries the charged groups of HS but also retains carbohydrate motifs, mostly presented as side chains on a synthetic polymer backbone. To date, only one example exists. Abdulsalam et al. recently generated a series of well-defined sGAG glycopolymer mimetics bearing glucosamine and glucuronic acid repeating disaccharides which were shown to effectively bind the S1 unit of the spike glycoprotein in a length and sulfation-dependent fashion.^31^ However, the mimetics examined in their work were shown to largely retain their anticoagulant activities, thus limiting their therapeutic potential.

In 2020, we published the synthesis of the first generation of sulfated glycooligomers and glycopolymers as sGAG mimetics and further demonstrated their ability to serve as broadband antivirals.^32^ We initially focused on targeting Human Papilloma Virus 16 (HPV16) given the importance of this virus in the development of invasive cancers, such as cervical cancer. Our experiments revealed that our sGAG mimetics could prevent HPV infection, both *in vitro* and *in vivo*. We then explored the generalizability of this approach. Additional studies with Herpes Simplex Virus (HSV), Influenza A Virus (IAV), and Merkel Cell Polyomavirus (MCPyV) showed that our compounds could also serve as broad-spectrum inhibitors of viral infection. Inspired by our work in this area, we chose to apply our strategy to design a library of sGAG glycopolymer mimetics with the potential to target SARS-CoV-2. In this study, we systematically varied structural parameters such as the linker connecting the carbohydrate and polymer scaffold (**GP-60-nl**), chain length (**GP-10** to **GP-300**), and density of carbohydrate side chains (**coGP-70** (**30, 50, and 70%**) and **coGP-300 (50%)**) (Figure 1). Polymers were synthesized by free-radical photopolymerization of carbohydrate monomers and also with *N*-hydroxyethylacrylamide (HEAA) as a comonomer by using 2,4,6-trimethylbenzoyldiphenyl phosphine oxide (TPO) as a photoinitiator. For polymerization, the mannose monomers were acetylated, according to a known protocol of Wilkins et al.^33^ We aimed to maximize the inhibitory potential of our sGAG mimetics by maximizing interactions between the glycopolymer and viral receptors. We hypothesized that longer chain sGAG glycopolymer mimetics would have increased inhibitory effects in comparison with their shorter chain counterparts. We also examined the sulfation density with the goal of reducing off-target effects and increasing therapeutic potential. Here, we hypothesized that it might be possible to reduce anticoagulant properties while maintaining high antiviral activity by tuning the charge density along the polymer backbone. Infection assays with SARS-CoV-2 revealed a “sweet spot” demonstrating for the first time how specific structural parameters could be used to maximize inhibition while minimizing anticoagulative properties.

**Figure 1:**
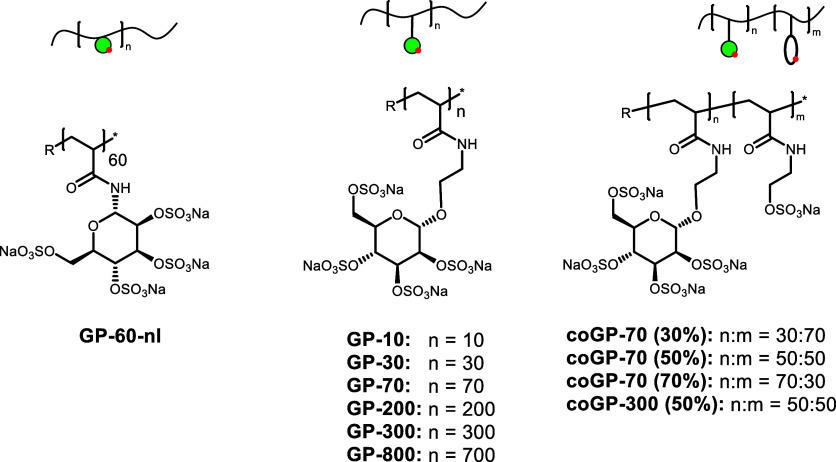
Glycopolymers designed and studied as inhibitors of SARS-CoV-2 infections.

## 2 Methods

### 2.1 Materials and Instrumentation

#### 2.1.1 Materials

Acetonitrile (99.9%, HPLC-grade), ammonium bicarbonate (99%), hydrochloric acid 1 M (HCl, p.a.), boron trifluoride diethyl etherate (BF_3_·Et_2_O, synth. grade), diethyl ether (p.a.), dichloromethane (99.9%, puriss., p.a.), d-(+)-mannose (99%), magnesium sulfate (MgSO_4_, 99.5%), trimethylamine (NEt_3_, 99%), sodium chloride (NaCl, 98%), sulfur trioxide trimethylamine complex (TMA·SO_3_, 95%), trifluoracetic acid (TFA 99%), and thiophenol (97%) were purchased from Sigma-Aldrich. Acryloyl chloride (97%) was purchased from Fisher Scientific GmbH. Dichloromethane (DCM, p.a.), dimethylformamide (DMF, 98%, for peptide synthesis), and ethyl acetate (analytical reagent grade) were purchased from ACROS Organics. Methanol (MeOH, p.a.), acetic anhydride (99.7%), and pyridine were purchased from VWR Chemicals. Diphenyl-(2,4,6-trimethylbenzoyl)-phosphine oxide (TPO, >98%) and *N*-hydroxyethylacrylamide (HEAA, >98%) were purchased from TCI chemicals.

#### 2.1.2 ^1^H NMR

^1^H NMR spectra were recorded at room temperature with Bruker AVANCE III 300 (for 300 MHz) and 600 (for 600 MHz) spectrometers. The chemical shifts were reported relative to solvent peaks (chloroform and water) as internal standards and reported as δ in parts per million (ppm). Multiplicities were abbreviated as s for singlet, d for doublet, t for triplet, and m for multiplet.

#### 2.1.3 Size-Exclusion Chromatography-Multiangle Light Scattering (H_2_O-SEC-MALS)

SEC analysis was conducted with an Agilent 1200 series HPLC system and three aqueous SEC columns provided by the Polymer Standards Service (PSS). The columns were two Suprema Lux analytical columns (8 mm diameter and 5 μm particle size) and one precolumn (50 mm, 2 × 160 Å of 300 mm and 1000 Å of 300 mm), allowing a theoretical detection of molar masses from 900 Da to 2000 kDa. The eluent was a buffer system consisting of Milli-Q water and 30% acetonitrile with 50 mM NaH_2_PO_4_, 150 mM NaCl, and 250 ppm of NaN_3_ with a pH = 7.0 (via addition of 50 mL 3 molar aqueous sodium hydroxide solution) filtered with an inline 0.1 μm membrane filter and running at 0.8 mL per minute. Measurements were recorded at room temperature. SEC calibration was performed using a BSA standard in 50 mM phosphate buffer. Multi-angle light scattering is recorded via mimDAWN TREOS and differential refractive index spectra with Optilab rEX both supplied by Wyatt Technologies EU. Data analysis was committed with Astra 5 software and a d*n*/d*c* value of 0.156 for each polymer.

#### 2.1.4 Size-Exclusion Chromatography

SEC analysis was conducted with an Agilent 1260 series HPLC system, one precolumn, and three aqueous SEC columns provided by GE Healthcare. The columns were three Suprema Lux analytical columns (100/100/1000). The eluent was a buffer system consisting of Milli-Q water with 10 mM PBS-buffer with pH = 7.4 and running at 1 mL per minute. Multi-angle light scattering is recorded via DAWN Heleos-II (Wyatt), λ = 660 nm, and differential refractive index spectra with Optilab T-rEX (Wyatt), λ = 660 nm, both supplied by Wyatt Technologies EU. Data analysis was performed with Astra software and a d*n*/d*c* value of 0.163 for each polymer.

#### 2.1.5 Freeze Dryer

Lyophilization was performed with an Alpha 1–4 LD instrument provided by Martin Christ Freeze-Dryers GmbH. A temperature of −42 °C and a pressure of 0.1 mbar were maintained throughout the freeze-drying process.

#### 2.1.6 Elemental Analysis

The ratios of carbon, hydrogen, nitrogen, and sulfur were determined using a Vario Micro Cube provided by Analysensysteme GmbH. The measurements were carried out by the Institute for Pharmaceutical and Medicinal Chemistry, Heinrich-Heine University Düsseldorf.

#### 2.1.7 High-Pressure Liquid Chromatography (HPLC)

RP-HPLC/MS (Reversed Phase-HPLC/Mass Spectroscopy) was performed on an Agilent Technologies 1260 Infinity System using an AT 1260 G4225A degasser, G1312B binary pump, G1329B automatic liquid sampler, G1316C thermostated column compartment, G1314F variable wavelength detector at 214 nm, and an AT 6120 quadrupole containing an electrospray ionization (ESI) source. The mobile phase consisted of buffer C (water:acetonitrile 95:5 (v/v), 0.1 vol % formic acid) and buffer D (water:acetonitrile 5:95 (v/v), 0.1 vol % formic acid). HPLC runs were performed on a Poroshell 120 EC-C18 (3.0 × 50 mm, 2.5 μm) RP column from Agilent at a flow rate of 0.4 mL/min of 95% buffer A and 5% buffer B (0–5 min), following a linear gradient to 100% buffer B (5–30 min) at 25 °C. ESI-MS for GlcNAc-oligomers and sulfates was performed using 95% buffer A and 5% buffer B without formic acid and a fragmentor voltage of 40–60 V (*m*/*z* range of 200 to 2000).

#### 2.1.8 Dynamic Light Scattering

DLS measurements were performed on a Zetasizer Nano ZS from Malvern. Samples were prepared by solving the polymers in PBS buffer (pH 7.4) with a concentration of 0.5 mg/mL. Before measurement, the samples were filtered through Whatman Puradisc 13 PTFE filters (5.0 mm, 13 diameter) from Cytiva. Measurements were performed in SARSTEDT polystyrene cuvettes.

### 2.2 Synthetic Methods

#### 2.2.1 Monosaccharide Synthesis

##### 2.2.1.1 M1 (**1**)

To synthesize monomer **M1**, commercially purchased mannose **1** (1 g, 5.5 mmol) was stirred with 0.88 g of ammonium bicarbonate (2 equiv) and magnesium sulfate for 72 h at 45 °C. The solution was then filtered and heated to 61 °C to decompose residual ammonium bicarbonate. By adding 1.56 g Di-*tert*-butyl dicarbonate (1.3 equiv) and stirring overnight, Boc-protected mannoseamine **3** precipitated and was filtered afterward. The sugar was then stirred with pyridine [10 mL/g] and acetic anhydride [10 mL/g] overnight, and after dilution with ethyl acetate extracted with 1 M HCl three times. After the Boc protecting group was removed by using 1:1 (v/v) TFA/DCM for 2 h at room temperature, compound **5** was isolated after evaporating the DCM and TFA under reduced pressure. Monomer **M1** was obtained by the reaction of **5** with acryloyl chloride. For this, 1.5 g of **5** (1 equiv, 4.5 mmol) was dissolved with 1.25 mL NEt_3_ (2.5 equiv) in DCM [10 mL/g], and the solution was cooled in an ice bath. 0.47 mL of acryloyl chloride (1.3 equiv) was then added, and the reaction was carried out at room temperature for 2 h. After extraction with NaHCO_3_, **M1** was purified by column chromatography (EE/Hexane 1:1 (v*:*v)). M1 was deprotected for RP-HPLC and ESI-MS measurements in an aqueous atmosphere. ^1^H NMR (600 MHz, CD_3_OD): δ (ppm) 6.50–6.27 (m, 2H), 5.75 (dd, *J* = 9.2, 2.8 Hz, 1H), 5.26 (d, *J* = 1.3 Hz, 1H).3.9–3.55 (m, 6H). ESI-MS *m*/*z*: calculated for C_9_H_15_NO_6_ [M + H]^+^ 234.09 and [M + Na]^+^ 256.08; found [M + H]^+^ 234.24 and [M + Na]^+^ 256.05.

##### 2.2.1.2 M2 (**2**)

The synthesis of the mannose monomer was adapted by Wilkins et al.^33^ The acetylated mannose acrylamide monomer was synthesized by dissolving d-mannose in a mixture of a 1:1 (v/v) mixture of pyridine/acetic anhydride [20 mL/g] and stirring at room temperature overnight. After diluting with ethyl acetate, the mixture was extracted three times with 1 M HCl solution. Evaporation of ethyl acetate resulted in 1,2,3,4,6-penta-*O*-acetyl-α-d-mannopyranose. One g of pentaacetylated mannose (1.0 equiv, 2.5 mmol) and 0.3 g of *N*-hydroxyethyl acrylamide (1.2 equiv, 3 mmol) were dissolved in DCM [2 mL/mmol] and flushed with argon gas for 10 min. Three mL of BF_3_·Et_2_O (10.0 equiv) was added through a syringe and the mixture was stirred at room temperature overnight. The reaction solution was washed three times with brine, and the organic phase was dried with MgSO_4_. The solvent was removed, which resulted in a pure acetylated monomer (AcO-ManAAm) with a relative purity of 98% and a yield of 78%. ^1^H NMR (300 MHz, CDCl_3_): δ (ppm) 2.00–2.16 (s, 12H, CH_3_ H1–4), 3.46–3.61 (m, 2H, CH_2_ H5), 3.79–4.02 (m, 2H, CH_2_, H6), 4.06–4.23 (m, 2H, CH_2_, H7), 4.82 (s, 1H, CH, H8), 5.22–5.69 (m, 4H, CH, H9–12), 6.15 (dd, ^2^ *J* = 10.2 Hz, ^3^ *J* = 17.1 Hz, 2H, CH_2_, H14), 6.32 (dd, ^2^ *J* = 1.2 Hz, ^3^ *J* = 17.1 Hz, 1H CH, H13) ESI-MS *m*/*z*: calculated for C_19_H_27_NO_11_ [M + H]^+^ 446.16 and [M + Na]^+^ 468.15; found [M + H]^+^ 446.46 and [M + H]^+^ 468.

#### 2.2.2 Polymer Synthesis

##### 2.2.2.1 GP60-nl (**3**)

100 mg portion of monomer **M1** (0.4 mmol) and 1.99 mg of 2,4,6-trimethylbenzoyldiphenyl phosphine oxide (TPO, 1.4 mol %, 0.0056 mmol) were dissolved in DMF [10 wt %] and the solution was flushed with Argon for 10 min and irradiated with UV-light (405 nm wavelength, with an intensity 45.2 mW/cm^2^). After an hour, the irradiation was stopped and 5 mL of NaOMe (0.2 M) in MeOH was added to the polymer solution and stirred for 1 h at room temperature. Solid matter had already precipitated and the residual solution was precipitated in diethyl ether. The precipitated polymer was dissolved in H_2_O, dialyzed against distilled H_2_O (three cycles, 2 kDa), and subsequently lyophilized. ^1^H NMR (600 MHz, D_2_O): δ [ppm] 7.79–7.59 (m), 7.00–6.99 (m), 5.31–4.89 (m, D_2_O overlapping), 4.01–3.15 (m), 2.32–1.33 (m).

##### 2.2.2.2 GP-10-OH (**4**)

222.7 mg of monomer **M2** (0.5 mmol) and 17.42 mg of TPO (10 mol %, 0.05 mmol) were dissolved in DMF [10 wt %], and the solution was flushed with Argon for 10 min and irradiated with UV-light (405 nm wavelength, with an intensity 45.2 mW/cm^2^). After an hour, the irradiation was stopped and 5 mL of NaOMe (0.2 M) in MeOH was added to the polymer solution and stirred for 1 h at room temperature. Solid matter had already precipitated and the residual solution was precipitated in diethyl ether. The precipitated polymer was dissolved in H_2_O, dialyzed against distilled water (three cycles, 2 kDa), and subsequently lyophilized. ^1^H NMR (600 MHz, D_2_O): δ [ppm] 7.79–7.59 (m), 7.00–6.99 (m), 5.00–4.83 (m, D_2_O overlapping), 4.04–3.27 (m), 1.42–1.32 (m).

##### 2.2.2.3 GP-30-OH (**5**)

222.7 mg of monomer **M2** (0.5 mmol) and 5.23 mg of TPO (3 mol %, 0.015 mmol) were dissolved in DMF [10 wt %] and the solution was flushed with Argon for 10 min and irradiated with UV-light (405 nm wavelength, with an intensity 45.2 mW/cm^2^). After an hour, the irradiation was stopped and 5 mL of NaOMe (0.2 M) in MeOH was added to the polymer solution and stirred for 1 h at room temperature. Solid matter had already precipitated and the residual solution was precipitated in diethyl ether. The precipitated polymer was dissolved in H_2_O, dialyzed against distilled H_2_O (three cycles, 2 kDa), and subsequently lyophilized. ^1^H NMR (600 MHz, D_2_O): δ [ppm] 4.93–4.88 (m, D_2_O overlapping), 4.04–3.25 (m), 2.37–1.38 (m).

##### 2.2.2.4 GP-70-OH (**6**)

222.7 mg of monomer **M2** (0.5 mmol) and 2.44 mg of TPO (1.4 mol %, 0.007 mmol) were dissolved in DMF [10 wt %], and the solution was flushed with Argon for 10 min and irradiated with UV-light (405 nm wavelength, with an intensity 45.2 mW/cm^2^). After an hour, the irradiation was stopped and 5 mL of NaOMe (0.2 M) in MeOH was added to the polymer solution and stirred for 1 h at room temperature. Solid matter had already precipitated and the residual solution was precipitated in diethyl ether. The precipitated polymer was dissolved in H_2_O, dialyzed against distilled water (three cycles, 2 kDa), and subsequently lyophilized. ^1^H NMR (600 MHz, D_2_O): δ [ppm] 4.85–4.77 (m, D_2_O overlapping), 3.98–3.11 (m), 2.19–1.22 (m).

##### 2.2.2.5 GP-200-OH (**7**)

445.4 mg portion of monomer **M2** (1 mmol) and 1.74 mg of TPO (0.5 mol %, 0.005 mmol) were dissolved in DMF [10 wt %] and the solution was flushed with Argon for 10 min and irradiated with UV-light (405 nm wavelength, with an intensity 45.2 mW/cm^2^). After an hour, the irradiation was stopped and 5 mL of NaOMe (0.2 M) in MeOH was added to the polymer solution and stirred for 1 h at room temperature. Solid matter had already precipitated and the residual solution was precipitated in diethyl ether. The precipitated polymer was dissolved in H_2_O, dialyzed against distilled water (three cycles, 2 kDa), and subsequently lyophilized. ^1^H NMR (600 MHz, D_2_O): δ [ppm] 5.00–4.83 (m, D_2_O overlapping), 4.1–3.21 (m), 2.35–1.38 (m).

##### 2.2.2.6 GP-300-OH (**8**)

445.4 mg of monomer **M2** (1 mmol) and 1.15 mg of TPO (0.33 mol %, 0.0033 mmol) were dissolved in DMF [10 wt %] and the solution was flushed with Argon for 10 min and irradiated with UV-light (405 nm wavelength, with an intensity 45.2 mW/cm^2^). After an hour, the irradiation was stopped and 5 mL of NaOMe (0.2 M) in MeOH was added to the polymer solution and stirred for 1 h at room temperature. Solid matter had already precipitated and the residual solution was precipitated in diethyl ether. The precipitated polymer was dissolved in H_2_O, dialyzed against distilled water (three cycles, 2 kDa), and subsequently lyophilized. ^1^H NMR (600 MHz, D_2_O): δ [ppm] 4.87–4.79 (m, D_2_O overlapping), 3.95–3.2 (m), 2.3–1.19 (m).

##### 2.2.2.7 GP-800-OH (**9**)

890.8 mg portion of monomer **M2** (2 mmol) and 0.87 mg of TPO (0.12 mol %, 0.0025 mmol) were dissolved in DMF [10 wt %] and the solution was flushed with Argon for 10 min and irradiated with UV-light (405 nm wavelength, with an intensity 45.2 mW/cm^2^). After an hour, the irradiation was stopped and 5 mL of NaOMe (0.2 M) in MeOH was added to the polymer solution and stirred for 1 h at room temperature. Solid matter had already precipitated and the residual solution was precipitated in diethyl ether. The precipitated polymer was dissolved in H_2_O, dialyzed against distilled water (three cycles, 2 kDa), and subsequently lyophilized. ^1^H NMR (600 MHz, D_2_O): δ [ppm] 5.00–4.83 (m, D_2_O overlapping), 4.07–3.24 (m), 2.3–1.32 (m).

##### 2.2.2.8 PHEAA-200-OH (**10**)

1151.3 mg of *N*-hydroxyethylacrylamide (10 mmol) and 1.74 mg TPO (0.5 mol %, 0.005 mmol) were dissolved in DMF [10 wt %] and the solution was flushed with Argon for 10 min and irradiated with UV-light (405 nm wavelength, with an intensity 45.2 mW/cm^2^). After 1 h, the irradiation was stopped, and the solution was precipitated in diethyl ether. The precipitated polymer was dissolved in H_2_O, dialyzed against distilled H_2_O (three cycles, 2 kDa), and subsequently lyophilized. ^1^H NMR (600 MHz, D_2_O): δ (ppm) 4.18–4.02 (m), 3.64–3.30 (m), 2.25–1.36 (m).

##### 2.2.2.9 coGP-70-OH (30%) (**11**)

133.6 mg of monomer M2 (0.3 mmol), 80.6 mg of HEAA (0.7 mmol), and 2.44 mg of TPO (1.4 mol %, 0.007 mmol) were dissolved in DMF [10 wt %] and the solution was flushed with Argon for 10 min and irradiated with UV-light (405 nm wavelength, with an intensity 45.2 mW/cm^2^). After an hour, the irradiation was stopped and 5 mL of NaOMe (0.2 M) in MeOH was added to the polymer solution and stirred for 1 h at room temperature. Solid matter had precipitated and the residual solution that was precipitated in diethyl ether was collected. The precipitated polymer was dissolved in H_2_O, dialyzed against distilled H_2_O (three cycles, 2 kDa), and subsequently lyophilized. ^1^H NMR (600 MHz, D_2_O): δ 7.79–7.59 (m), 7.00–6.99 (m), 4.82–4.80 (m, D_2_O overlapping), 3.93–3.04 (m), 2.20–1.17 (m).

##### 2.2.2.10 coGP-70-OH (50%) (**12**)

222.7 mg of monomer M2 (0.5 mmol), 57.56 mg of HEAA (0.5 mmol), and 2.44 mg of TPO (1.4 mol %, 0.007 mmol) were dissolved in DMF [10 wt %] and the solution was flushed with Argon for 10 min and irradiated with UV-light (405 nm wavelength, with an intensity 45.2 mW/cm^2^). After an hour, the irradiation was stopped and 5 mL of NaOMe (0.2 M) in MeOH was added to the polymer solution and stirred for 1 h at room temperature. Solid matter had precipitated and the residual solution that was precipitated in diethyl ether was collected. The precipitated polymer was dissolved in H_2_O, dialyzed against distilled water (three cycles, 2 kDa), and subsequently lyophilized. ^1^H NMR (600 MHz, D_2_O): δ [ppm] 7.79–7.59 (m), 7.00–6.99 (m), 4.82–4.80 (m, D_2_O overlapping), 3.93–3.04 (m), 2.20–1.17 (m).

##### 2.2.2.11 coGP-70-OH (70%) (**13**)

311.8 mg of monomer M2 (0.7 mmol), 34.54 mg of HEAA (0.3 mmol), and 2.44 mg TPO (1.4 mol %, 0.007 mmol) were dissolved in DMF [10 wt %] and the solution was flushed with Argon for 10 min and irradiated with UV-light (405 nm wavelength, with an intensity 45.2 mW/cm^2^). After an hour, the irradiation was stopped and 5 mL of NaOMe (0.2 M) in MeOH was added to the polymer solution and stirred for 1 h at room temperature. Solid matter had precipitated and the residual solution that was precipitated in diethyl ether was collected. The precipitated polymer was dissolved in H_2_O, dialyzed against distilled water (three cycles, 2 kDa), and subsequently lyophilized. ^1^H NMR (600 MHz, D_2_O): δ [ppm] 4.82–4.80 (m, D_2_O overlapping), 3.95–3.13 (m), 2.34–1.29 (m).

##### 2.2.2.12 coGP-300-OH (50%) (**14**)

445.4 mg of monomer M2 (1 mmol), 115.13 mg of HEAA (1 mmol), and 2 mg of TPO (0.33 mol %, 0.006 mmol) were dissolved in DMF [10 wt %], and the solution was flushed with argon for 10 min and irradiated with UV-light (405 nm wavelength, with an intensity 45.2 mW/cm^2^). After an hour, the irradiation was stopped and 5 mL of NaOMe (0.2 M) in MeOH was added to the polymer solution and stirred for 1 h at room temperature. Solid matter had precipitated and the residual solution that was precipitated in diethyl ether was collected. The precipitated polymer was dissolved in H_2_O, dialyzed against distilled water (three cycles, 2 kDa), and subsequently lyophilized. ^1^H NMR (600 MHz, D_2_O): δ [ppm] 4.82–4.80 (m, D_2_O overlapping), 3.97–3.10 (m), 2.34–1.31 (m).

#### 2.2.3 General Sulfation Protocol

Sulfation of glycopolymers and glycol copolymers was performed as in an earlier published protocol.^2^ TMA·SO_3_ (40 equiv per OH-group) was used as a sulfating agent and dissolved with the polymer in DMF and stirred for 18 h at 70 °C. After the solution was cooled down to room temperature, 20 equiv of aqueous sodium acetate solution (20%) was added for quenching at 0 °C. The solvent mixture was evaporated under a reduced pressure, dialyzed (MWCO 5–10 kDa), and lyophilized. The degree of sulfation was determined via an elemental analysis. Theoretical values were calculated for 100% sulfation (without considering the end groups), and the degree of sulfation was calculated from the obtained values. For this purpose, the S/C ratio was calculated for optimal (100%) sulfation and the S/C ratio was calculated for actual sulfation. ((S/C)_actual_/(S/C)_optimal_) × 100 forms the actual degree of sulfation.

##### 2.2.3.1 GP60-nl (**3S**)

^1^H NMR (600 MHz, D_2_O): δ [ppm] 5.51–3.68 (m, D_2_O overlapping), 1.98–1.42 (m). Elemental analysis: theoretical values (*n* = 60): % C = 17.19; % H = 1.76; % N = 2.17; % S = 19.88; measured values (*n* = 60): % C = 21.40; % H = 3.52; % N = 2.67; % S = 16.74.

##### 2.2.3.2 GP-10 (**4S**)

^1^H NMR (600 MHz, D_2_O): δ [ppm] 5.38–5.13 (m), 4.49–2.80 (m, D_2_O overlapping), 2.50–1.4 (m). Elemental analysis: theoretical values (*n* = 10): % C = 21.12; % H = 2.33; % N = 1.99; % S = 18.19; measured values (*n* = 10): % C = 19.49; % H = 3.62; % N = 1.94; % S = 13.50.

##### 2.2.3.3 GP-30 (**5S**)

^1^H NMR (600 MHz, D_2_O): δ 5.29–5.18 (m), 5.01–3.23 (m, D_2_O overlapping), 2.40–1.23 (m). Elemental analysis: theoretical values (*n* = 30): % C = 19.27; % H = 2.21; % N = 2.04; % S = 18.71; measured values (*n* = 30): % C = 18.48; % H = 3.34; % N = 2.28; % S = 16.57.

##### 2.2.3.4 GP-70 (**6S**)

^1^H NMR (600 MHz, D_2_O): δ [ppm] 5.30–5.18 (m), 5.09–3.22 (m, D_2_O overlapping), 2.38–1.38 (m). Elemental analysis: theoretical values (*n* = 70): % C = 19.27; % H = 2.21; % N = 2.04; % S = 18.71; measured values (*n* = 70): % C = 17.23; % H = 3.34; % N = 1.74; % S = 15.51.

##### 2.2.3.5 GP-200 (**7S**)

^1^H NMR (600 MHz, D_2_O): δ [ppm] 5.30–5.18 (m), 5.05–3.19 (m, D_2_O overlapping), 2.46–1.12 (m). Elemental analysis: theoretical values (*n* = 200): % C = 19.27; % H = 2.21; % N = 2.04; % S = 18.71; measured values (n = 200): % C = 17.56; % H = 3.11; % N = 1.96; % S = 15.28.

##### 2.2.3.6 GP-300 (**8S**)

^1^H NMR (600 MHz, D_2_O): δ [ppm] 5.32–5.21 (m), 5.04–3.14 (m, D_2_O overlapping), 2.50–1.22 (m). Elemental analysis: theoretical values (*n* = 300): % C = 19.27; % H = 2.21; % N = 2.04; % S = 18.71; measured values (n = 300): % C = 17.71; % H = 3.28; % N = 1.74; % S = 16.20.

##### 2.2.3.7 GP-800 (**9S**)

^1^H NMR (600 MHz, D_2_O): δ [ppm] 5.30–5.18 (m), 5.02–3.11 (m, D_2_O overlapping), 2.35–1.23 (m). Elemental analysis: theoretical values (*n* = 800): % C = 19.27; % H = 2.21; % N = 2.04; % S = 18.71; measured values (n = 800): % C = 18.31; % H = 2.87; % N = 2.05; % S = 15.21.

##### 2.2.3.8 PHEAA-200 (**10S**)

^1^H NMR (600 MHz, D_2_O): δ (ppm) 4.18–4.03 (m), 3.62–3.33 (m), 2.25–1.36 (m). Elemental analysis: theoretical values (*n* = 200): % C = 27.65; % H = 3.72; % N = 6.45; % S = 14.76; measured values (n = 200): % C = 23.59; % H = 3.94; % N = 5.16; % S = 11.49.

##### 2.2.3.9 coGP-70 (30%) (**11S**)

^1^H NMR (600 MHz, D_2_O): δ [ppm] 5.27–5.17 (m), 4.96–3.21 (m, D_2_O overlapping), 2.42–1.31 (m). Elemental analysis: theoretical values (*n* = 21, *m* = 49): % C = 22.84; % H = 2.85; % N = 3.92; % S = 17.03; measured values (n = 21, m = 49): % C = 20.91; % H = 4.04; % N = 3.41; % S = 13.43.

##### 2.2.3.10 coGP-70 (50%) (**12S**)

^1^H NMR (600 MHz, D_2_O): δ [ppm] 5.27–5.17 (m), 4.98–3.04 (m, D_2_O overlapping), 2.42–1.24 (m). Elemental analysis: theoretical values (*n* = 35, *m* = 35): % C = 21.29; % H = 2.58; % N = 3.1; % S = 17.76; measured values (n = 35, m = 35): % C = 19.71; % H = 3.58; % N = 2.71; % S = 14.05.

##### 2.2.3.11 coGP-70 (70%) (**13S**)

^1^H NMR (600 MHz, D_2_O): δ [ppm] 5.29–5.19 (m), 5.03–3.24 (m, D_2_O overlapping), 2.33–1.35 (m). Elemental analysis: theoretical values (*n* = 46, *m* = 19): % C = 20.24; % H = 2.39; % N = 2.55; % S = 18.25; measured values (n = 46, m = 19): % C = 17.98; % H = 3.7; % N = 2.19; % S = 15.81.

##### 2.2.3.12 coGP-300 (50%) (**14S**)

^1^H NMR (600 MHz, D_2_O): δ [ppm] 5.17–5.08 (m), 5.03–3.11 (m, D_2_O overlapping), 2.24–1.12 (m). Elemental analysis: theoretical values (*n* = 150, *m* = 150): % C = 21.29; % H = 2.57; % N = 3.1; % S = 17.76; measured values (n = 150, m = 150): % C = 18.24; % H = 3.69; % N = 2.46; % S = 15.29.

### 2.3 Biological Assays

#### 2.3.1 VSV-ΔG+G Virus Production

Selection of BHK-G43 was performed in GMEM-5% FBS containing 0.5 mg/mL Hygromycin B and 1 mg/mL Zeocin. Selected cells were stimulated to express the vesicular stomatitis virus (VSV)-G protein by addition of 1 nM Mifepristone into fresh GMEM-5% FBS (cells kept at 37 °C/5% CO_2_/6 h). Cells were then overnight infected with VSV-ΔG+G, which is a VSV that genetically lacks its G protein gene and contains a coding sequence for a green fluorescent protein (GFP) and luciferase (a kind gift from PD Dr. Gert Zimmer, Institute of Virology and Immunology, Mittelhäusern, Switzerland). Supernatants were collected, centrifuged at 200 g/5 min, aliquoted, and frozen at −80 °C. Later, the newly produced VSV-ΔG+G virus was titrated on Vero cells by serial dilution in DMEM-10% FBS and 1h infection at 37 °C in addition to 1× wash and 37 °C/5% CO_2_/16–18 h incubation in new DMEM-10% FBS media. GFP-positive cells were counted the next day under the fluorescent microscope (Zeiss Axiovert 200M, Zeiss, Oberkochen, Germany) (titers expressed as fluorescent focus units per milliliter, FFU/mL).

#### 2.3.2 SARS-CoV-2 Pseudovirus (PsV) Preparation

SARS-CoV-2 PsV is a replication-defective VSV, which carries the SARS-CoV-2 S protein on its surface as the sole glycoprotein used for the cell entry.^34^ For production, about 1 × 10^7^ HEK293TT cells were seeded into 100 mm culture dishes about 19 h prior to experimentation. Cells were transfected with 12 μg of a plasmid carrying the Wuhan SARS-CoV-2 S protein sequence (YP_009724390.1, pCG1-SARS-2-S) with a C-terminal truncation (i.e., deletion of the last 21 amino acids in the S protein) pCG1-SARS-2-Sd21 ^35^ using 36 μL Transit-LT1 (Mirus) per plate according to the manufacturer’s instructions. Subsequently, the transfection medium was replaced with 3 mL of DMEM containing VSVΔG+G at a multiplicity of infection (MOI) of 0.03 FFU/cell. After incubation for 1 h at 37 °C, the plate was washed once with the medium and incubated with an anti-VSV G protein antibody (obtained from I1-Hybridoma cells) for 30 min at 37 °C (to neutralize potential still existing VSV-ΔG+G viruses). Cells were washed twice and incubated for 18–22 h at 37 °C (each step included fresh DMEM-10% FBS). The following day, the supernatant containing SARS-CoV-2 PsV was cleared from the cells and cell debris by brief centrifugation. Subsequently, the virus was concentrated using ultrafiltration (Amicon 100 kDa, Merck # UFC9100024) and finally titrated on Vero cells to obtain FFU/mL titer. The virus inoculum was stored at −80 °C.

#### 2.3.3 SARS-CoV-2 PsV Infection

About 6000 Vero E6 cells per well were seeded into 96-well optical bottom plates and maintained overnight in DMEM-10% FBS at 37 °C and 5% CO_2_. SARS-CoV-2 PsV was incubated with the indicated concentrations of heparin and glycomimetic polymers at room temperature for 1 h in 10 mM HEPES (pH 7.4)/NaCl (150 mM) prior to infection of Vero E6 cells. For infection, Vero E6 cells were washed once with PBS and infected (MOI of 5 FFU/cell) with preincubated virus solutions for 1h at 37 °C and 5% CO_2_, respectively. Subsequently, the inoculum was removed from cells and replaced with a growth medium, and cells were incubated for 24 h at 37 °C and 5% CO_2_. After fixation in 4% paraformaldehyde, and staining of cell nuclei with RedDot, plates were analyzed for GFP-expressing (infected) cells by automated microscopy and image analysis as previously described.^36^

#### 2.3.4 SARS-CoV-2 Preparation

The human SARS-CoV-2 virus hCoV-19/Germany/FI1103201/2020 isolate (EPI-ISL_463008) with the D614G mutation in its S protein was prepared by propagation on Vero cells (less than 5 passages).^35^

#### 2.3.5 SARS-CoV-2 Infection

SARS-CoV-2 was incubated with the indicated concentrations of heparin and glycomimetic polymers at room temperature for 1 h before the infection of Vero E6 cells. As a control, the virus was incubated with HEPES solution (pH 7.4) without substances. For infection, Vero E6 cells were washed with PBS and infected (MOI of 0.001) with preincubated virus solutions for 1h at 37 °C and 5% CO_2_, respectively. After the 1 h infection, inoculums were removed from cells and replaced with a plaque medium (minimal essential medium (MEM)) containing 0.42% BSA, 1 mM l-glutamine, 20 mM HEPES, 0.24% NaHCO_3_, 200 IU/mL penicillin, 0.2 mg/mL streptomycin, 2% FBS, and 0.7% oxoid agar and cells were incubated for 72 h at 37 °C and 5% CO_2_. After removal of the agar, virus plaques were visualized by Coomassie blue dye staining (Roth, Karlsruhe, Germany, Brilliant blue #R250, dissolved in a methanol/acetic acid/distilled water mixture). Virus titers were indicated as plaque forming units (PFUs)/mL.

#### 2.3.6 Activated Partial Thromboplastin Time (aPTT), Thrombin Clotting Time (TCT), and anti-Xa-Activity

Selected polymers were dissolved in PBS buffer (phosphate-buffered buffered saline, pH = 7.4) at concentrations of 11, 55, 110, and 165 μg/mL. An aliquot of 270 μL of solution was added to 2.7 mL of citrate-anticoagulated venous human blood to obtain final concentrations of 1, 5, 10, and 15 μg/mL per measurement. Blood without addition was used to determine the normal values. All measurements were performed as triplicates. After centrifugation, the aPTT and TCT for each plasma sample were measured on a Sysmex CS 5100 system (provided by Siemens Healthineers, Germany).

## 3 Results and Discussion

### 3.1 Design and Synthesis of sGAG Glycopolymer Mimetics

sGAG glycopolymer mimetics are composed of a synthetic backbone decorated with sulfated carbohydrate motifs, thereby retaining two key features of their natural analogues: the carbohydrate motifs and sulfate groups. This simplified structure allows for straightforward synthesis from polymerizable carbohydrate motifs, so-called glycomonomers, by free or controlled radical polymerization, followed by global sulfation, and can be accessed at high molecular weights e.g., similar to natural HS.

sGAG glycopolymer mimetics in this study were synthesized following previously established protocols starting from tailor-made glycomonomers exclusively focusing on mannose as the carbohydrate motif, as shown in Scheme 1. Acrylamide groups were introduced at the anomeric position as polymerizable groups. For this study, two different monomers were synthesized by varying the linker between the polymerizable unit and the mannose: **M1**, a mannose acrylamide monomer with no additional linker, and **M2** containing a *N*-hydroxyethylacrylamide with an ethyl linker between the acrylamide unit and the anomeric center of the mannose. Both monomers were then applied in free-radical photopolymerizations. To avoid unwanted side reactions during polymerization, mannose monomer hydroxyl groups were acetyl-protected. The polymerization was performed by using a 405 nm LED and TPO as a photoinitiator. After polymerization, glycopolymers were deacetylated by treatment with sodium methanolate. Both monomers were successfully homopolymerized; however, **M1** showed much lower yield and molecular weights indicating that this monomer is not well suited for radical polymerization, likely due to steric effects given its proximity to the acrylamide group. Therefore, we continued the synthesis of our small library of homopolymers using **M2**. Conditions were tuned to selectively vary the chain length from 10 to 800 (**GP-10-OH** to **GP-800-OH**). In addition, **M2** was copolymerized with *N*-hydroxyethylacrylamide (HEAA) at two different chain lengths (**coGP-70** and **coGP-300**) with varying ratios of mannose/HEAA (from 30 to 50 to 70% mannose). All intermediate glycopolymers were characterized by aqueous SEC-MALS and ^1^H NMR (see Supporting Information for the spectra). Finally, glycopolymers were globally sulfated using a previously established protocol.^30^ Degree of sulfation was measured by elemental analysis, and successful sulfation was further confirmed by 1H NMR (see Supporting Information). In total, 11 sGAG glycopolymer mimetics (GPs) and their according nonsulfated precursors were isolated (Table 1). As an additional control compound without a carbohydrate motif, HEAA was homopolymerized (**PHEAA-200-OH**) and globally sulfated giving **PHEAA-200**.

**Scheme 1:**
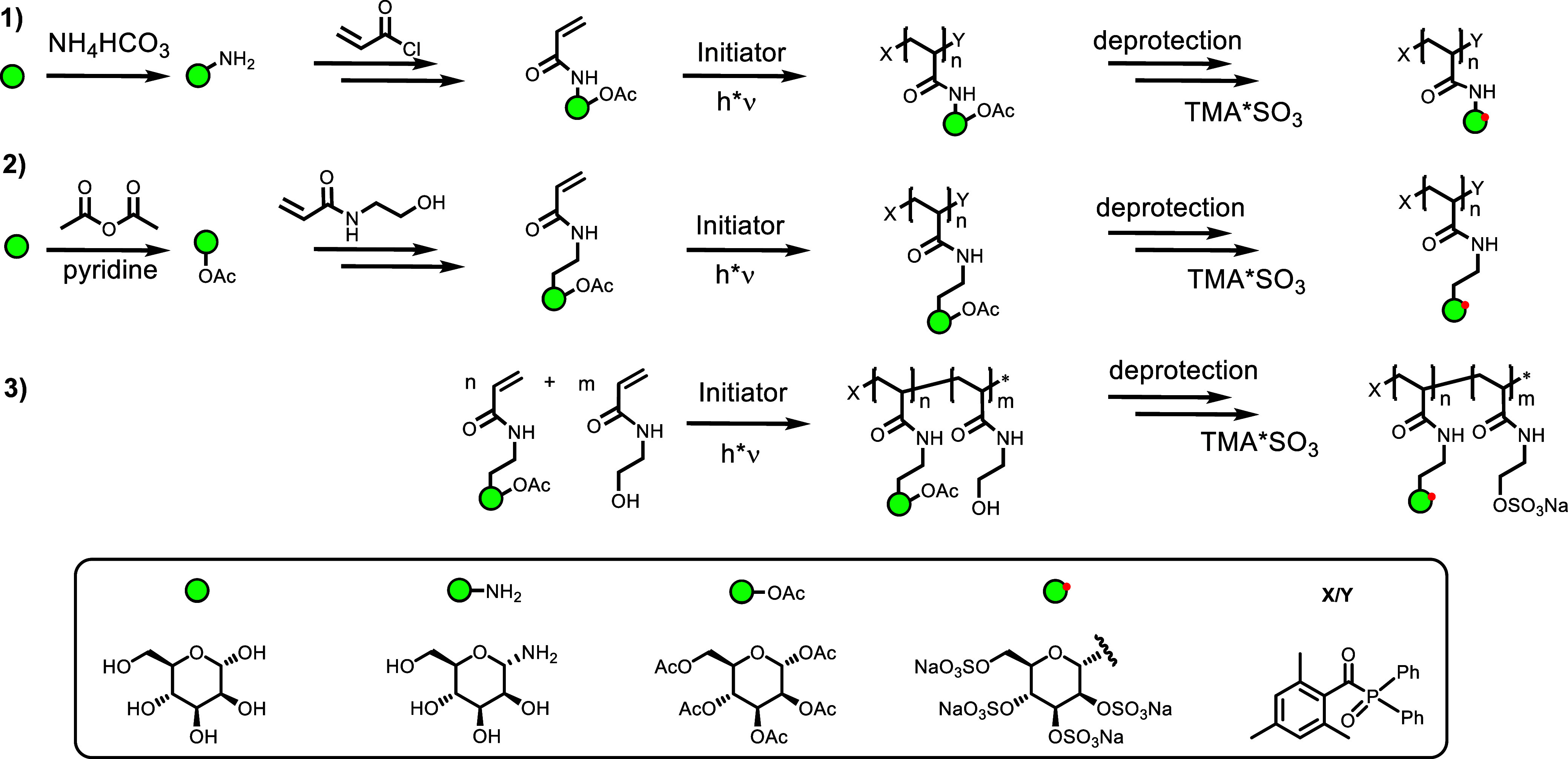
Synthesis of Glycomonomers and Homo- and Coglycopolymers and Their Global Sulfation^a^

a (1) Homopolymers from a glycomonomer with no linker: polymerization (1 equiv M1, 1.4 mol % TPO, DMF [10 wt %], 1 h *hv* (μ = 405 nm, 45.2 mW/cm^2^), deprotection (NaOMe/MeOH (0.2 M), 1 h, rt), sulfation (40 equiv/OH TMA·SO_3_, 18 h, 70 °C); (2) homopolymers from a glycomonomer with an ethyl linker: polymerization (1 equiv M2, *x* mol % TPO; for *x*, see Supporting Information), DMF [10 wt %], 1 h *hv* (μ = 405 nm, 45.2 mW/cm^2^), deprotection (NaOMe/MeOH (0.2 M), 1 h, rt), sulfation (40 equiv/OH TMA·SO_3_, 18 h, 70 °C); (3) copolymers from a glycomonomer with an ethyl linker and HEAA: polymerization (1 equiv M2, *x* equiv HEAA; for *x*, see Supporting Information), 1.4 mol % TPO, DMF [10 wt %], 1 h *hv* (μ = 405 nm, 45.2 mW/cm^2^), deprotection (NaOMe/MeOH (0.2 M), 1 h, rt), sulfation (40 equiv/OH TMA·SO_3_, 18 h, 70 °C).

**Table 1:**
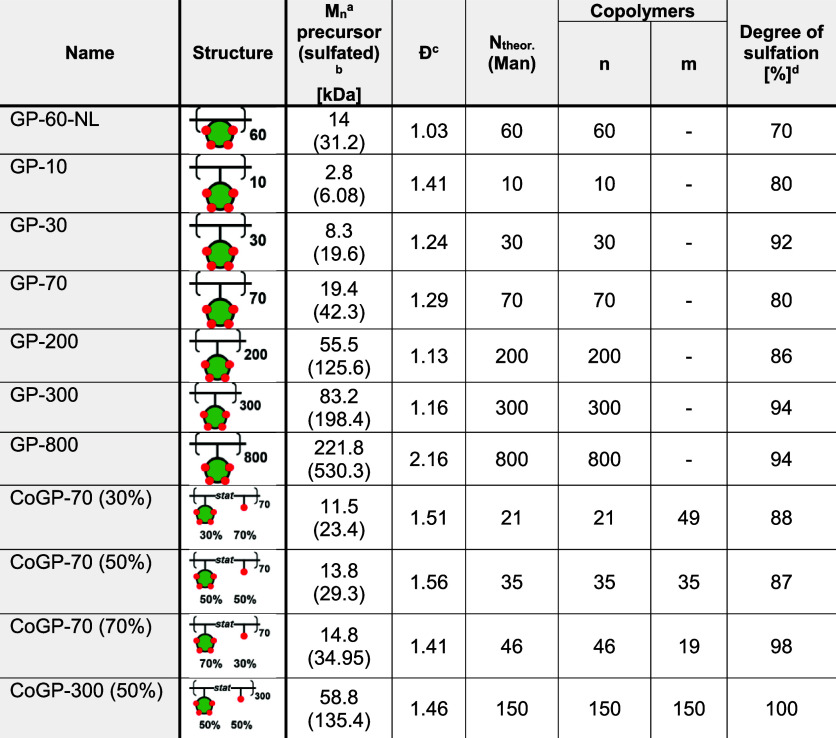
Overview of the Structural Parameters of the Precursor and Sulfated Glycopolymers^f^

a *M*_n_ determined by aqueous SEC-MALLS.

b *M*_n_ determined by calculation based on the degree of sulfation.

c *D̵* determined by aqueous SEC-MALLS.

d Degree of sulfation determined by elemental analysis via an S/C ratio (for detail, see Supporting Information), n.d. = not determined.

f Found in HR-ESI-MS.

g Nomenclature gives the degree of polymerization, e.g., GP-10, and for the non-sulfated precursor, OH is added, e.g., GP-10-OH. Copolymers (coGP) additionally carry the information of the ratio of Mannose/HEAA as the theoretical (number) percentage of mannose monomers, e.g., coGP-70 (30%).

We based our polymer design on previous structure property correlations, e.g., it has been shown for both natural sGAGs and sGAG mimetics that chain length strongly impacts their antiviral and anticoagulant properties.^37^ While high-molecular-weight HP is the more potent inhibitor of virus adhesion, it also shows increased anticoagulant properties in comparison with its lower molecular weight fragments. In pharmaceutical applications of HP as an anticoagulant, unfractionated heparin shows variable dose–response relationships due to its structural heterogeneity and requires close monitoring during administration.^38^ Furthermore, side-effects such as heparin-induced thrombocytopenia may also be observed.^39^ Therefore, typically, fractionated lower-molecular-weight HP is used instead of unfractionated heparin.

A structural parameter that has been less studied is the positioning and related density of the sulfate groups along the sGAG chain. For example, HS consists of segments with high, low, or no sulfation.^40,41^ For natural sGAGs, it is highly challenging to analyze or even control such segments of sulfation. For glycopolymers, this can be more readily controlled through the density of carbohydrate motifs along the polymer chain, e.g., in a copolymer with noncarbohydrate monomers where segments with no carbohydrate side chains represent nonsulfated or lower sulfated segments. Indeed, for other types of glycopolymers, e.g., as inhibitors of bacterial adhesion, it has been shown that the density of carbohydrate motifs can strongly impact their binding affinity (or avidity) and thus their biological activity.^42–47^ Surprisingly, it is not the highest density and thus the highest number of carbohydrates that lead to the highest activity, but it is often a reduced density that leads to optimal binding. One reason for this finding is likely the steric crowding in glycopolymers that are too densely decorated with carbohydrate side chains, which limits the accessibility of the carbohydrates in binding, e.g., to a protein receptor.

Here, we systematically investigate the effect of carbohydrate density and thus sulfate density on SARS-CoV-2 inhibition using the first series of sGAG glycopolymer mimetics. We also explore how differences in the linker length (**M1** vs **M2**) influenced carbohydrate accessibility and thus SARS-CoV-2 inhibition. Initially, we examined hydrodynamic radii using dynamic light scattering experiments, as shown in Figure 2. It can be expected that a larger hydrodynamic radius indicates a larger and less densely coiled polymer structure in solution and thus would afford higher accessibility to bind, e.g., to the viral capsid proteins. As expected, with an increase in the chain length, the hydrodynamic radii increase from **GP-10** to **GP-300**. Interestingly, when retaining the same degree of polymerization and thus chain length (DP 70) while reducing the number of mannose units by replacing them with HEAA, a decrease in the hydrodynamic radius was observed. We tentatively explain this by a lower number and density of sulfate groups and thus less intramolecular electrostatic repulsion whereby the polymer can adapt to a more coiled conformation.^48,49^

**Figure 2:**
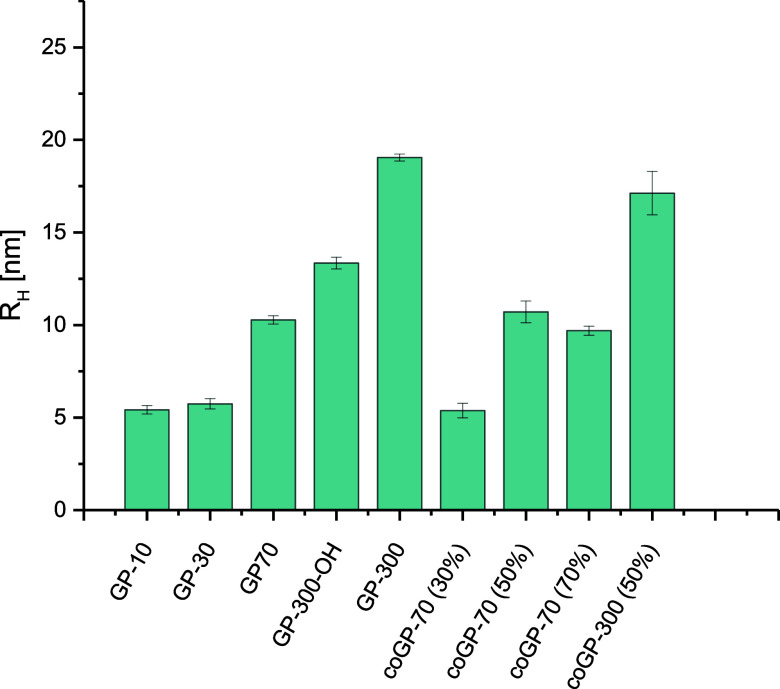
Hydrodynamic radii for selected compounds determined by DLS in PBS buffer: **GP-10**, **GP-30**, **GP-70**, **GP-300-OH**, **GP-300**, **coGP-70** (30%, 50%, and 70%), and **coGP-300** (50%).

### 3.2 GAG Mimetics Inhibit SARS-CoV-2 Pseudotyped Virus Infection

With our library of sGAG glycopolymer mimetics and controls in hand, we investigated their biological activity as inhibitors of virus entry and tested their anticoagulant properties. To investigate the inhibitory potential of sGAG glycopolymer mimetics, we initially used Vesicular Stomatitis Virus pseudotyped with the SARS-CoV-2 spike protein (SARS-CoV-2 PsV). Since SARS-CoV-2 exhibits only limited propagation in cell culture and is therefore difficult to purify, the SARS-CoV-2 PsVs have been widely used to investigate the cell attachment to receptors and entry.^28^ Here, we preincubated SARS-CoV-2 PsV with sGAG glycopolymer mimetics at indicated concentrations and subsequently infected the Vero cells (Figure 3A). As a positive control, we made use of HP and carrageenan, two polysaccharides that are known to interfere with attachment of a variety of viruses by occupying the heparan sulfate-binding sites in the viral surface proteins in a concentration-dependent manner.^10,11,50^ Indeed, both sulfated polysaccharides reduced the SARS-CoV-2 PsV infection of Vero cells in a dose-dependent manner (Figure 3B). Infection was reduced to a negligible level already at concentrations of 0.1 mg/mL, whereas carrageenan exhibited a slight yet significant higher potency to interfere with infection at 0.01 mg/mL compared to HP.

**Figure 3:**
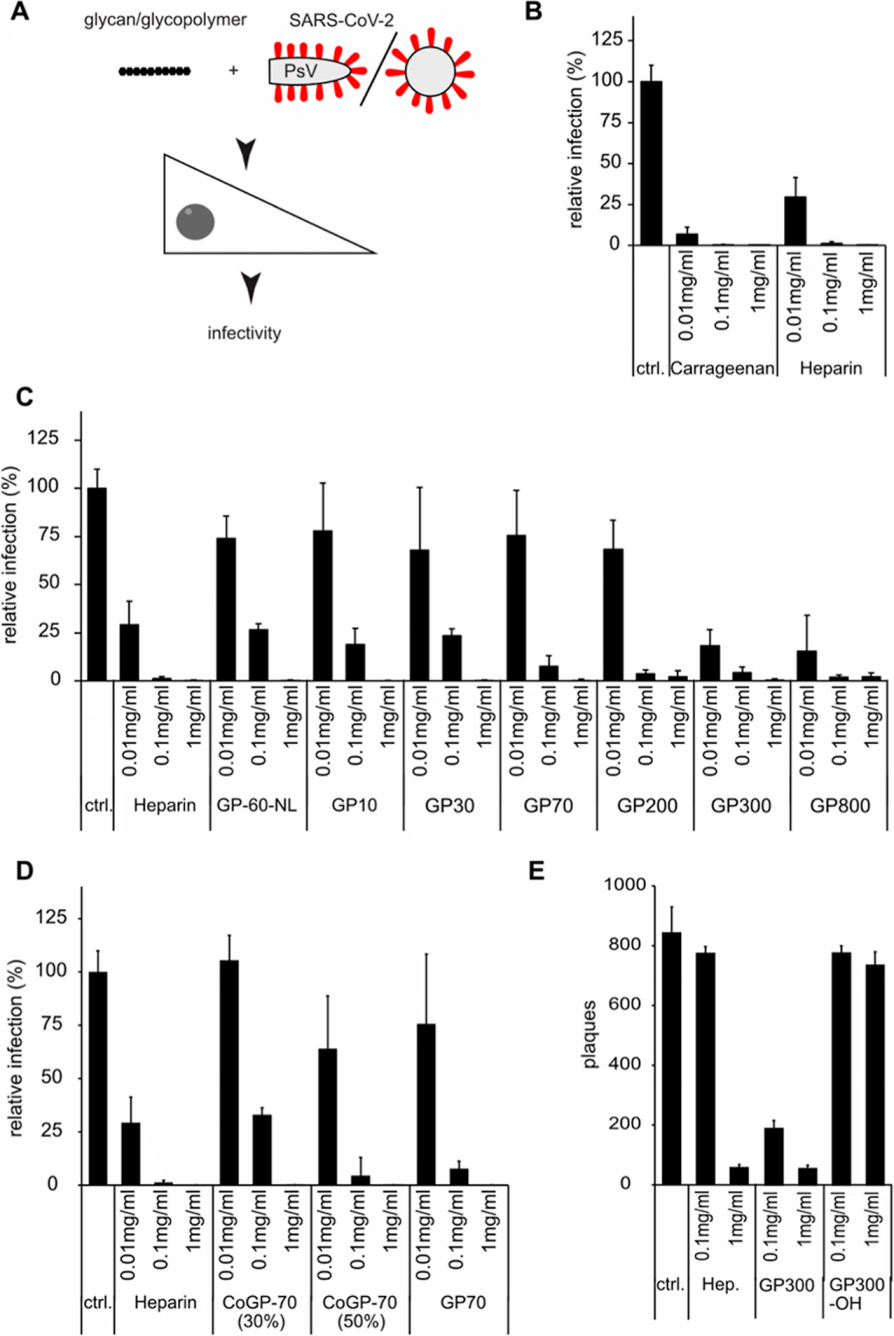
Inhibitory potential of glycomimetic compounds on SARS-CoV-2 infection. (A) Schematic depiction of the experimental procedure. (B–D) SARS-CoV-2 PsVs were incubated with glycosaminoglycans or glycomimetic polymers at the indicated concentrations for 1 h. Subsequently, this mixture was added to cells for 1 h, after which the inoculum was replaced by a growth medium. Next, cells were fixed 24 h post infection (p.i.) and stained with RedDot for nucleus detection. The number of GFP-expressing (infected) cells was determined by automated microscopy and image analysis, normalized to the untreated control, and displayed as relative infection ±standard deviation (SD). (E) SARS-CoV-2 was incubated with glycosaminoglycans or glycomimetic polymers at the indicated concentrations for 1 h. Subsequently, this mixture was added to cells for 1 h, after which the inoculum was replaced by a plaque medium. About 72 h p.i. plaques were visualized by Coomassie blue staining. The number of plaques was counted and displayed as plaque forming units ±SD.

Notably, when the various sGAG glycopolymer mimetics were tested in parallel experiments, all mimetics, independent of their degree of polymerization, interfered with infection at concentrations ≥0.1 mg/mL, and at concentrations of 1 mg/mL, all GP abrogated infection (Figure 3C). There was, however, a clear tendency that mimetics with a higher degree of polymerization exhibited a higher efficacy to inhibit infection at lower concentrations. GP > 30 abrogated infections at concentrations of 0.1 mg/mL, whereas GPs > 200 were as efficient as HP in interfering with infection. The inhibitory effect required the presence of sulfation, as the nonsulfated control polymers of **GP-70-OH**, **GP-200-OH**, **GP-300-OH**, and **GP-800-OH** failed to affect infection even at the highest concentrations (Figure S42). Overall, our data suggest that even lower molecular weight sGAG glycopolymer mimetics had the potency to bind to HS sites on the SARS-CoV-2 spike protein, with a clear tendency that longer polymers with more sulfated sugars have a stronger efficacy like that of natural HP. Finally, we observed that **GP-60-NL** had slightly reduced effects on infection compared to **GP-70** (Figure 3C). While both contain a similar number of sugars, but different linker lengths, it may be that a longer linker as in **GP-70** allows for better engagement of the sulfated sugar to the spike protein.

Next, we investigated whether the degree of sulfated sugars and thus the density of sulfate groups on sGAG glycopolymer mimetics would play a major role in engaging the SARS-CoV-2 spike protein, thus interfering with infection. Using **GP-70** in comparison to **coGP-70 (30%)** and **coGP-70 (50%)**, we observed no major difference in the degree of sulfation on the inhibitory potential (Figure 3D). However, lower degrees of sulfation were revealed to have a slightly reduced inhibitory potential, e.g., **GCoP-70 (30%)** to **GP-70**. This indicates that while sulfated sugars are key to the inhibitory potential of sGAG glycopolymer mimetics, their number and thus density may be less important. Taken together, our data indicated that the length of the polymer is more important for engagement of the SARS-CoV-2 spike protein than the number and density of sulfated sugars or the linker length.

### 3.3 sGAG Glycopolymer Mimetics as Inhibitors of SARS-CoV-2

Our data indicated that sGAG glycopolymer mimetics can engage the SARS-CoV-2 spike protein on VSV-pseudotyped particles, thereby suggesting that they may be able to reduce the burden of SARS-CoV-2 infections. To investigate whether this ability can be replicated for actual SARS-CoV-2 infections, we used a SARS-CoV-2 isolate from Germany with a D614G mutation in the S protein that is propagated in Vero cells. Here, SARS-CoV-2-containing supernatants from infected Vero cells were incubated with selected sGAG glycopolymer mimetics for 1 h, subsequently added to cells for another 1 h, after which the inoculum was replaced with an overlay and the resulting plaques from spreading infections into neighboring cells were quantified. As a control, we again employed HP. The amount of HP or sGAG mimetic polymers to reduce the number of plaques to a similar extent than SARS-CoV-2 PsV infection was about ten times higher, likely a result of HP and sGAG polymer-engaging serum proteins of cell supernatants (Figure 3E). However, in correlation with our SARS-CoV-2 PsV infection data, SARS-CoV-2 plaque formation was clearly reduced by **GP-300**, while unsulfated **GP-300-OH** had no effect on infection. It is noteworthy that **GP-300** was more potent in attenuating SARS-CoV-2 plaque formation than HP, perhaps due to the presence of serum proteins in the inoculum that could be more prone to engage HP rather than sGAG mimetic polymers.

### 3.4 Anticoagulant Properties of sGAG Glycopolymer Mimetics

HP has been used as an anticoagulant drug for many decades.^51^ However, this property limits its use as antiviral due to undesired side effects.^52,53^ Notably, it exerts its anticoagulative activity via the activation of antithrombin (AT), a serine protease inhibitor that can inhibit different clotting factors, especially clotting factors Xa (FXa) and IIa (FIIa), the latter of which is also called thrombis.^54^ The inhibition of the two clotting factors occurs via different mechanisms. While FXa is mainly inhibited by an allosteric activation of AT triggered by a specific pentasaccharide sequence,^55^ FIIa is inhibited by the so-called template effect^56^ in which the long HP chains allow the formation of a complex of AT and FIIa. A routine laboratory test used to characterize the anticoagulant properties of HP as well as sGAG glycopolymer mimetics is the activated partial thromboplastin time (aPTT) and the thrombin clotting time (TCT) which generally measure clotting times.^57,58^ The aPTT monitors the intrinsic and the common final pathway of the coagulation cascade which includes among others the activity of FXa and FIIa.^57^ Therefore, a prolonged aPTT indicates an inhibition of protease factors in the intrinsic and common coagulation pathway. In contrast, the TCT measures the time needed to convert fibrinogen to fibrin, which is activated by thrombin (FIIa). Therefore, an increased TCT can indicate an inhibition of thrombin as would be expected with the administration of HP.^57^

Based on previous findings for other sGAG glycopolymer mimetics,^59–62^ we hypothesized that the density of sulfate groups on the polymer chain might influence anticoagulant properties. In antiviral studies, we have seen that a reduction in sulfate density, while retaining high molecular weight, can still afford highly potent viral inhibitors. Thus, a simultaneous reduction in anticoagulant properties would be an important step forward in developing sGAG glycopolymer mimetics for antiviral treatments. To investigate the blood compatibility of our sGAG glycopolymer mimetics, we selected a subset of structure varying in sulfation density along the polymer backbone for aPTT testing. This included GP-70, CoGP-70 (30%), CoGP-70 (50%), CoGP-70 (70%), and HEAAP-200. The results of the aPTT measurement are shown in Figure 4A. aPTT values for untreated serum samples were 27 s, approximately.

**Figure 4:**
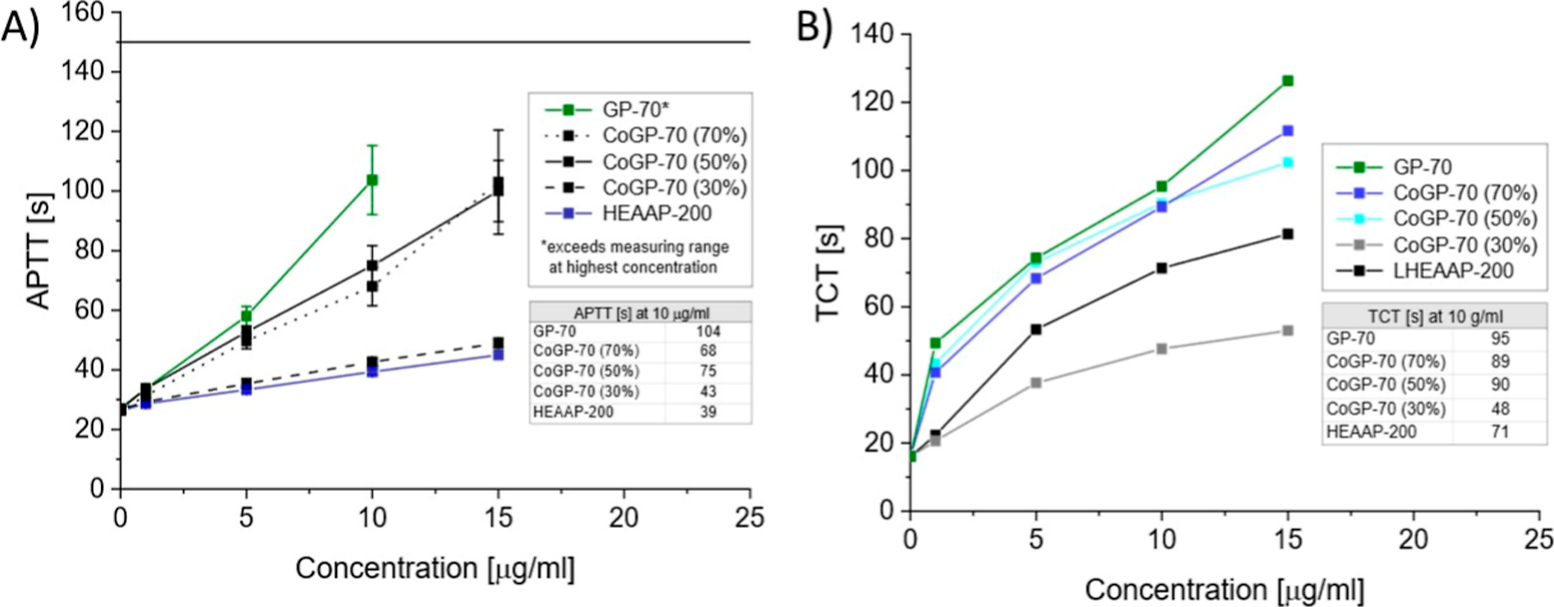
Anticoagulant properties for polymers **GP-70**, **coGP-70 (30%)**, **coGP-70 (50%)**, **coGP-70 (70%)**, and **HEAAP-200** at 0, 5, 10, and 15 μg/mL in human plasma: (A) aPTT (values for untreated serum samples were around 27 s); (B) TCT (values for untreated serum samples were around 16 s).

Considering first only the influence of the mannose and thus sulfate density, while maintaining a uniform DP of 70, aPTT is hardly influenced at 10 μg/mL by addition of **CoGP-70 (30%)**, the compound with the lowest density. **HEAAP-200** presenting a single sulfate group per repeating unit and thus a lower sulfate density, yet at a higher DP of 200, shows similar aPTT values. With increasing mannose content from **CoGP-70 (50%)** to **CoGP-70 (70%)**, we observed prolonged aPTT of 68 and 75 s, respectively. For the homopolymer, **GP-70**, the increase is even more pronounced, and accordingly, the aPTT at a concentration of 15 μg/mL is already above the detection limit (150 s). In the therapeutic administration of heparin, typical aPTT values are obtained between 50 to 80 s. At a concentration of 10 μg/mL, which corresponds to the lowest concentration used in the antiviral assays, only **CoGP-70 (30%)** is below this threshold.

In addition to aPTT, TCT was measured, which gives the conversion of fibrinogen to fibrin by FIIa (thrombin) (Figure 4B). The lowest TCT is observed for the glycopolymer with the lowest mannose density. Increase of mannose and thus sulfate density leads to an increase in TCT. While in the aPTT test, HEAAP showed very similar results as **coGP-70 (30%)**, for the TCT, it shows significantly higher values yet still below the values of the sGAG glycopolymer mimetics with a higher mannose content (**coGP-70 (50%)** and **coGP-70 (70%)**). Finally, the anti-FXa activity of the sGAG glycopolymer mimetics prepared in this study was measured. Interestingly, no activity could be detected for any of the samples. As expected, nonsulfated glycopolymers showed no influence on aPTT or TCT activity, as well as no anti-FXa activity. Taken together, these studies reveal, for the first time, how rationally designed sGAG glycopolymer mimetics can maintain a high inhibitory potential against SARS-CoV-2 while minimizing off-target effects such as anticoagulation.

## 4 Conclusion

In conclusion, in this work, we introduced a library of sGAG glycopolymer mimetics as potential inhibitors of SARS-CoV-2. Our rationally designed library was constructed to examine two key parameters: (i) the influence of the chain length and (ii) the effects of sulfate density on viral inhibition. Parallel studies involving Vesicular Stomatitis Virus pseudotyped with the SARS-CoV-2 spike protein (SARS-CoV-2 PsV) revealed that all mimetics, independent of their degree of polymerization, interfered with infection at concentrations ≥0.1 mg/mL; at concentrations of 1 mg/mL, all mimetics abrogated infection. As expected, sGAG glycopolymer mimetics with a higher degree of polymerization exhibited a higher efficacy, inhibiting infection at lower concentrations. Notably, comparable results were observed for select mimetics in infection assays conducted with SARS-CoV-2 (Wuhan).

Further studies examining the influence of sulfation density revealed that longer glycopolymer-based sGAG mimics with a similar degree of polymerization but with different sulfation densities (e.g., **GP-70** vs **coGP-70 (30%)** and **coGP-70 (50%)**) had a similar impact on the inhibitory potential in studies with SARS-CoV-2 PsV at concentrations of 1 mg/mL. However, sGAG mimetic with **CoGP-70 (30%)** with a lower sulfation density exhibited a significant reduction in the anticoagulant activity with aaPT values measuring 43 s at 10 μg/mL in comparison to GP-70 (104 s at 10 μg/mL) and heparin (50–80 s at 10 μg/mL). Notably, the measured aPTT was just above those obtained for untreated serum samples (27 s at 10 μg/mL).

Taken together, these studies reveal that for sGAG mimetic glycopolymers, chain length affects the inhibition of SARS-CoV-2 attachment for both pseudovirus and authentic virus infections, and that above a critical chain length, the density of carbohydrate and sulfate groups can be reduced, maintaining their high antiviral activity while minimizing their anticoagulant activity. The results demonstrate, to the best of our knowledge, the first example of a rationally designed sGAG glycopolymer mimetic with a high inhibitory potential and limited anticoagulant activity. This work is especially important because it provides a platform for designing novel sGAG mimetics with reduced side effects while maintaining a high therapeutic potential. Our current efforts are focused on exploring the potential of these compounds to serve as broad-spectrum viral inhibitors for other viruses known to engage sGAGs.

## Supplementary Material



## Notes

Detailed experimental procedures, additional figures and tables, and additional spectral data (e.g., NMR, SEC, HPLC, ESI-MS, EA, and DLS where appropriate) for the characterization of all new compounds (PDF)
