# Supplementary material for: Controlling the Sulfation Density of Glycosaminoglycan Glycopolymer Mimetics Enables High Antiviral Activity against SARS-CoV‑2 and Reduces Anticoagulant Activity
Source: Biomacromolecules. 2025 Jul 7;26(8):5169–81. doi: 10.1021/acs.biomac.5c00576 (PMC12344700; doi:10.1021/acs.biomac.5c00576)
Supplement: Supplementary file 1 [file bm5c00576_si_001.pdf]

Controlling the sulfation density of sulfated glycosaminoglycan glycopolymer mimetics enables high antiviral activity against SARS-CoV-2 and reduces anticoagulant activity

Miriam Hoffmann[a,#], Lorand Bonda[a,#], Ines Fels[b], Darisuran Anhlan[c], Eike Hrincius[c], Derik Hermsen[d], Stephan Ludwig[c], Mario Schelhaas[b]*, Nicole L. Snyder[e]* and Laura Hartmann[a,f]*

[a] Department of Organic and Macromolecular Chemistry, Heinrich-Heine-University Düsseldorf, Universitätsstraße 1, 40225 Düsseldorf, Germany.

[b] Institute of Cellular Virology, ZMBE and Cells in Motion Interfaculty Centre CiMIC, University of Münster, Münster 48149, Germany.

[c] Institute of Molecular Virology, ZMBE, University of Münster, Münster 48149, Germany.

[d] Central Institute of Laboratory Medicine, Medical Faculty, University Hospital Düsseldorf, Heinrich-Heine-University, Düsseldorf, Germany

[e] Department of Chemistry, Davidson College, Davidson, North Carolina 28035, United States.

[f] Institute for Macromolecular Chemistry, University of Freiburg, Stefan-Meier-Str. 31, 79104 Freiburg i.Br., Germany.

#These authors contributed equally.

*Email: schelhaas@uni-muenster.de (for virus-related queries), and nisnyder@davidson.edu and laura.hartmann@makro.uni-freiburg.de (for synthesis-related queries)

Supporting Information

Monomer Synthesis

Monomer M1 (1)

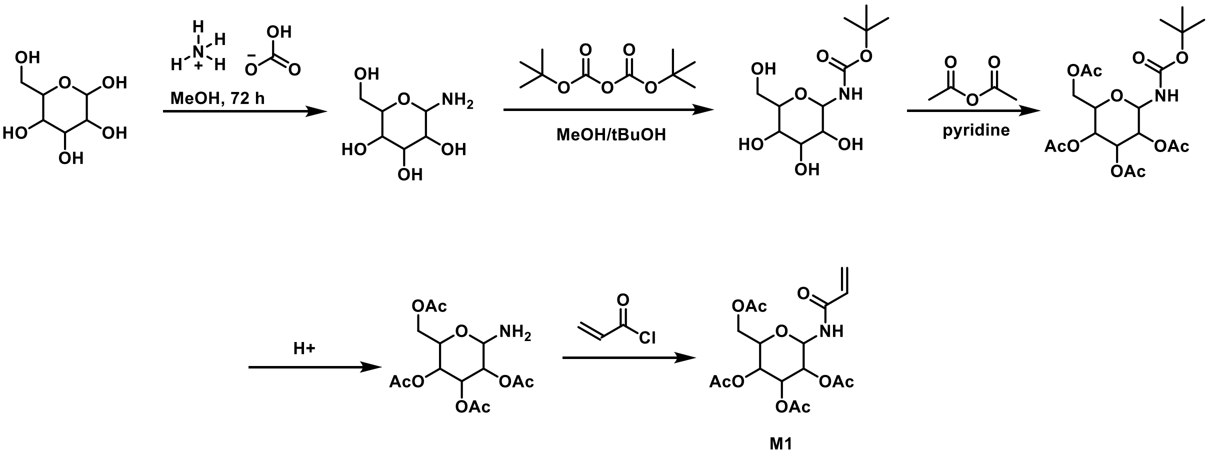

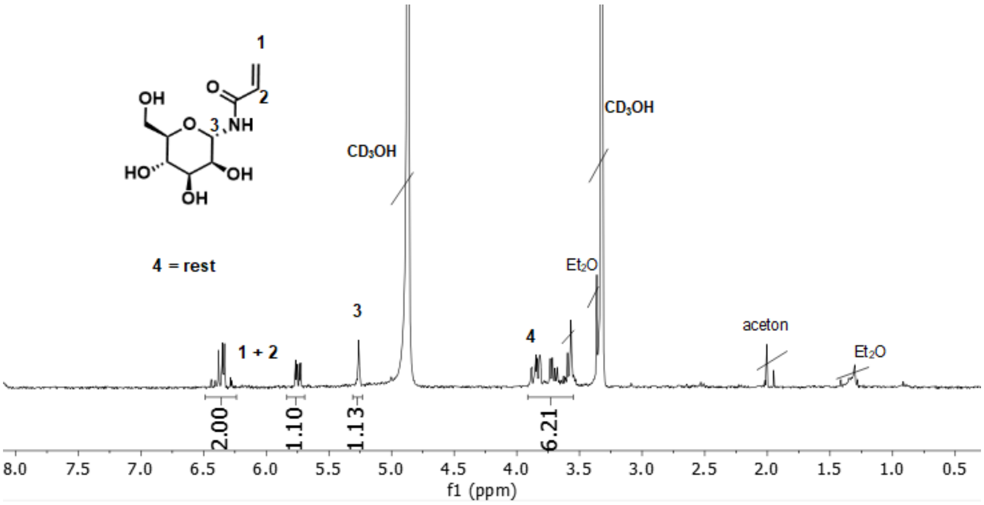

Figure S1. ^{1}H NMR spectrum (600 MHz, CD_3OD) of M1: δ (ppm) 6.50 – 6.27 (m, 2H), 5.75 (dd, J = 9.2, 2.8 Hz, 1H), 5.26 (d, J = 1.3 Hz, 1H).3.9-3.55 (m, 6H).

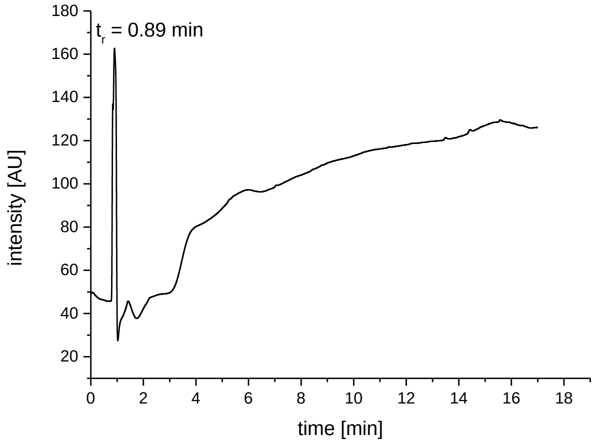

Figure S2. RP-HPLC of M2 (A: 95% H_2O/ 5% MeCN/ 0.1% Formic Acid; 100% A -> 50% A in 30 min): t_r = 0.89 min.

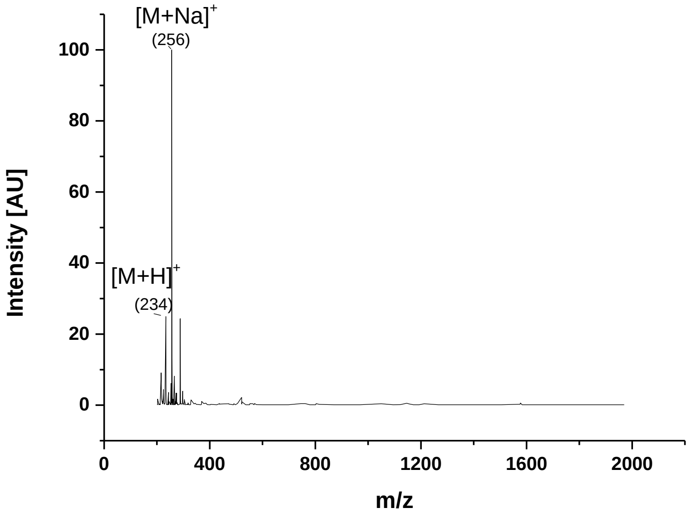

Figure S3. ESI-MS of M1.

Monomer M2 (2)

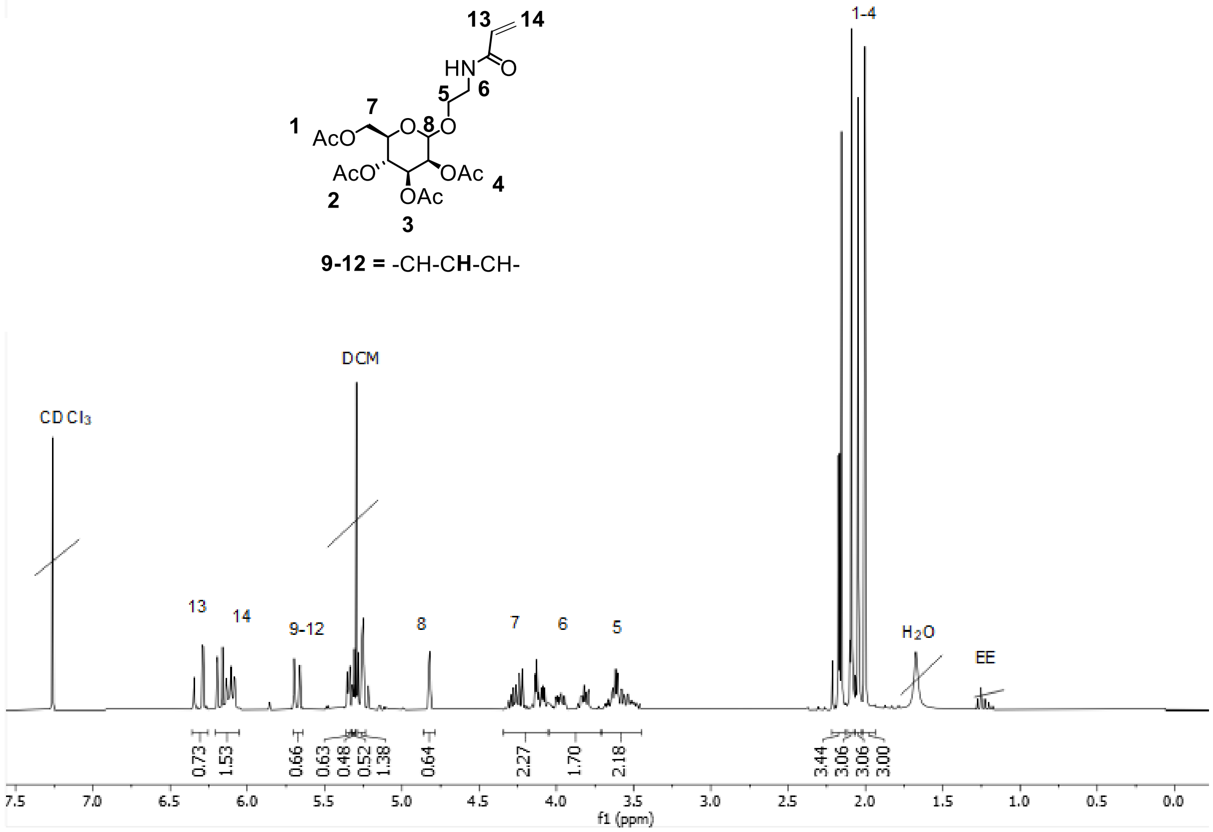

Figure S4. ^{1}H NMR (300 MHz, $CDCl_3$) spectrum of M2: δ (ppm) 2.00-2.16 (s, 12H, C**H**$_3$ H1-4), 3.46-3.61 (m, 2H, C**H**$_2$ H5), 3.79-4.02 (m, 2H, C**H**$_2$, H6), 4.06-4.23 (m, 2H, C**H$_2$**, H7), 4.82 (s, 1H, C**H**, H8), 5.22-5.69 (m, 4H, C**H**, H9-12), 6.15 (dd, ^{2}J=10.2 Hz, ^{3}J=17.1 Hz, 2H, C**H**$_2$, H14), 6.32 (dd, ^{2}J=1.2 Hz, ^{3}J=17.1 Hz, 1H C**H**, H13)

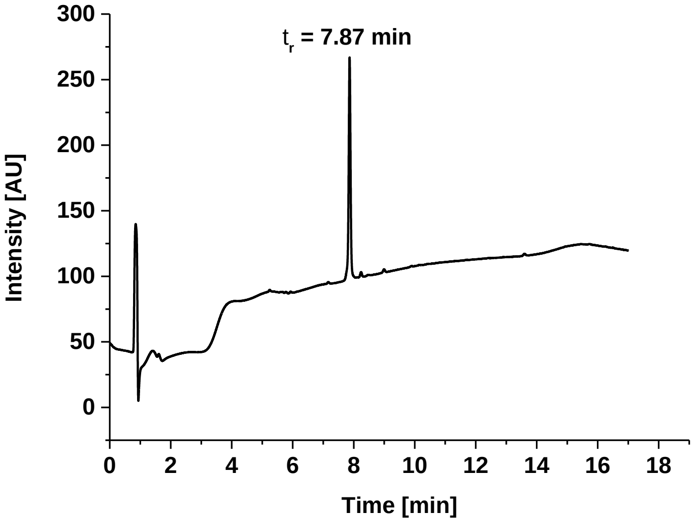

Figure S5. RP-HPLC of M2 (A: 95% H_2O/ 5% MeCN/ 0.1% Formic Acid; 100% A -> 50% A in 30 min): t_r = 7.87 min.

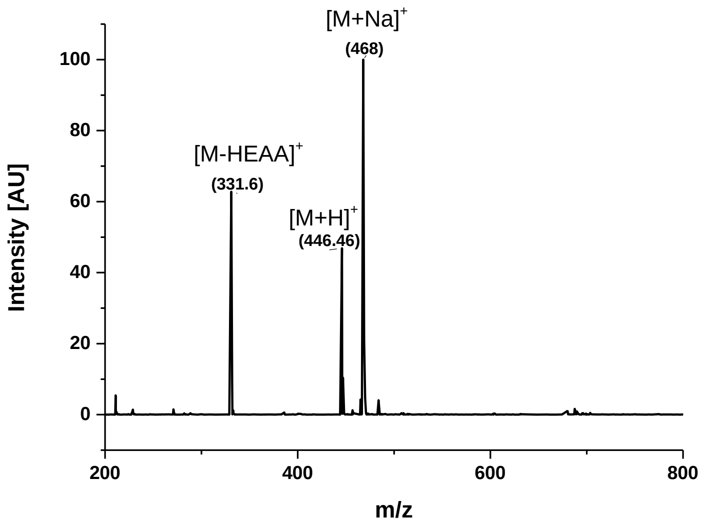

Figure S6. ESI-MS of M2.

GP60-nl (3)

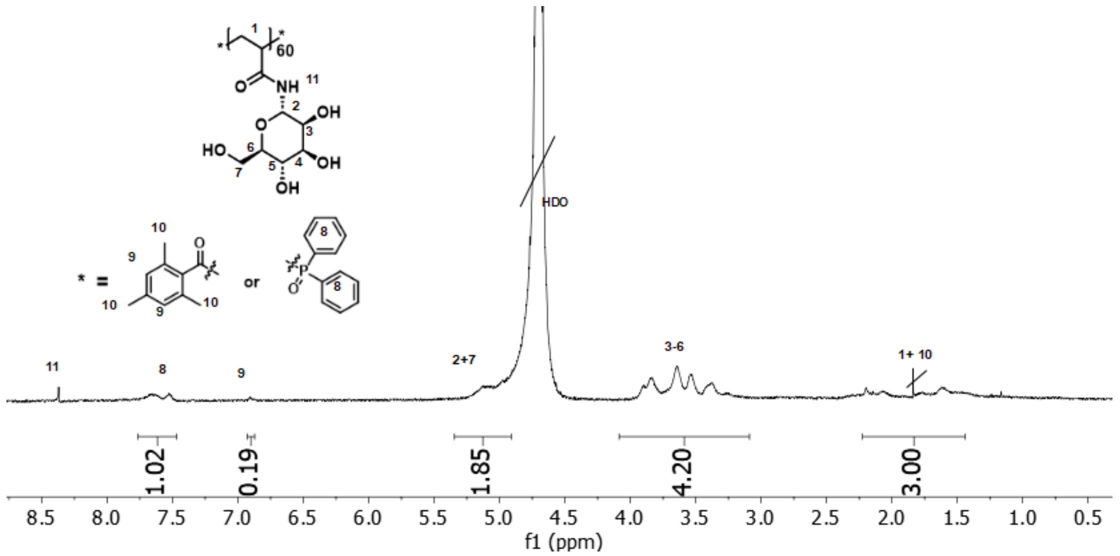

Figure S7. ^{1}H NMR spectrum (600 MHz, D_2O) of GP-60-OH-nl: δ [ppm] 7.79-7.59 (m, **8**), 7.00-6.99 (m, **9**), 5.31-4.89 (m, **2+7,** D_2O overlapping), 4.01-3.15 (m, **3-6**), 2.32-1.33 (m, **1+10**).

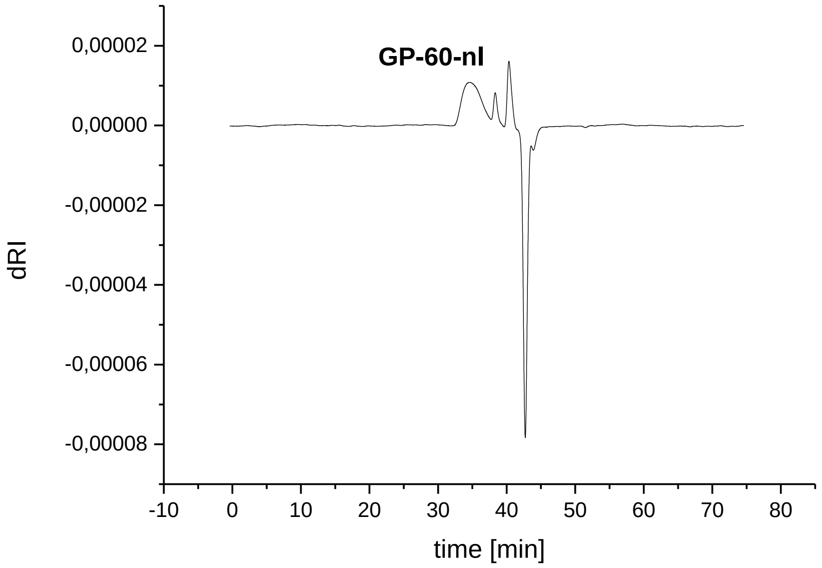

Figure S8. H_2O-SEC spectrum of GP-60-OH-nl.

GP-10-OH (4)

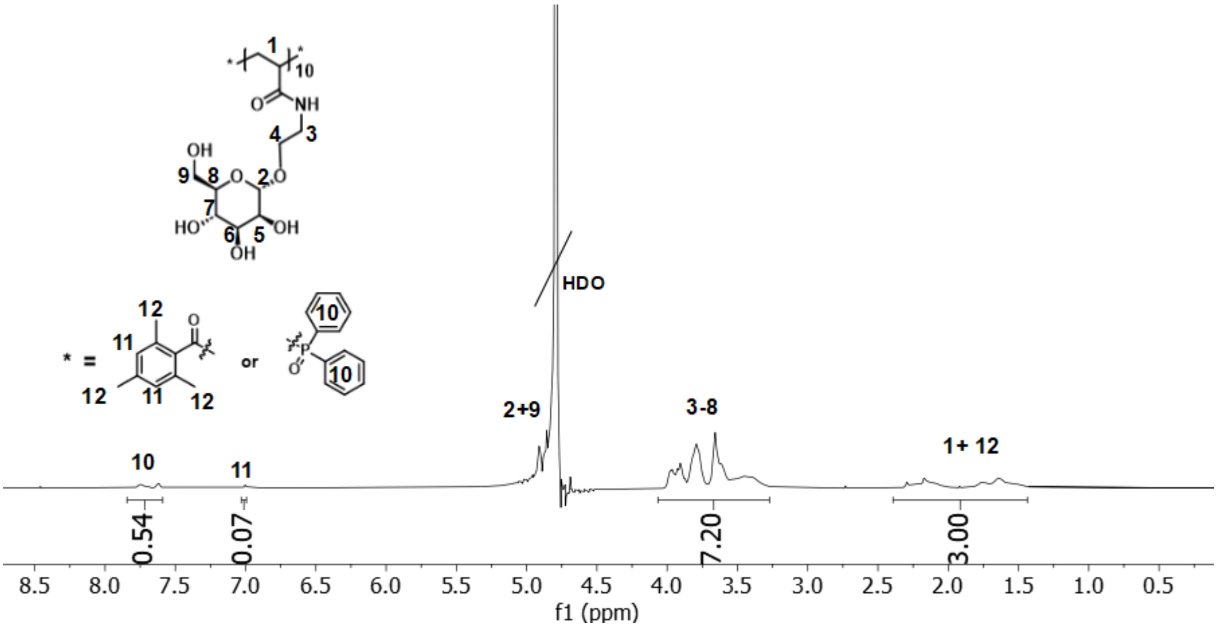

Figure S9. ^{1}H NMR spectrum (600 MHz, D_2O) of GP-10-OH: δ [ppm] 7.79-7.59 (m, **10**), 7.00-6.99 (m, **11**), 5.00-4.83 (m, **2+9,** D_2O overlapping), 4.04-3.27 (m, **3-8**), 1.42-1.32 (m, **1+12**).

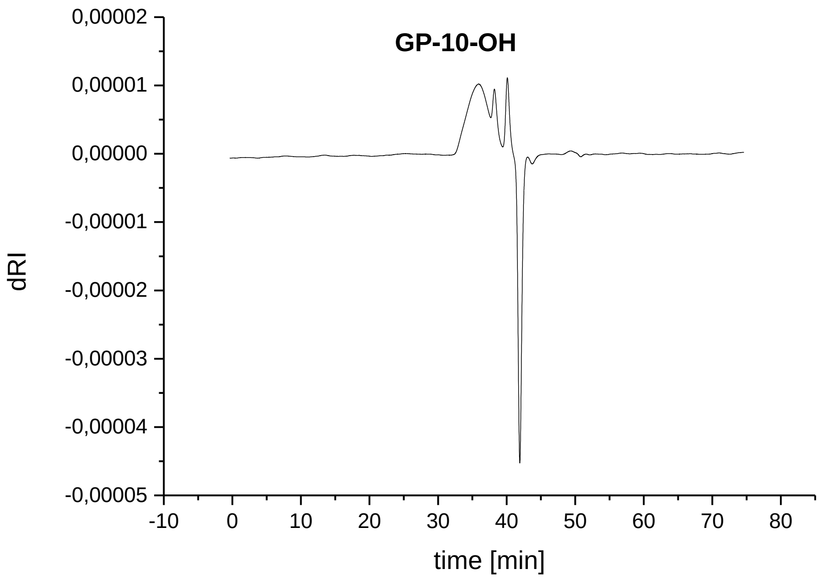

Figure S10. H_2O-SEC spectrum of GP-10-OH.

GP-30-OH (5)

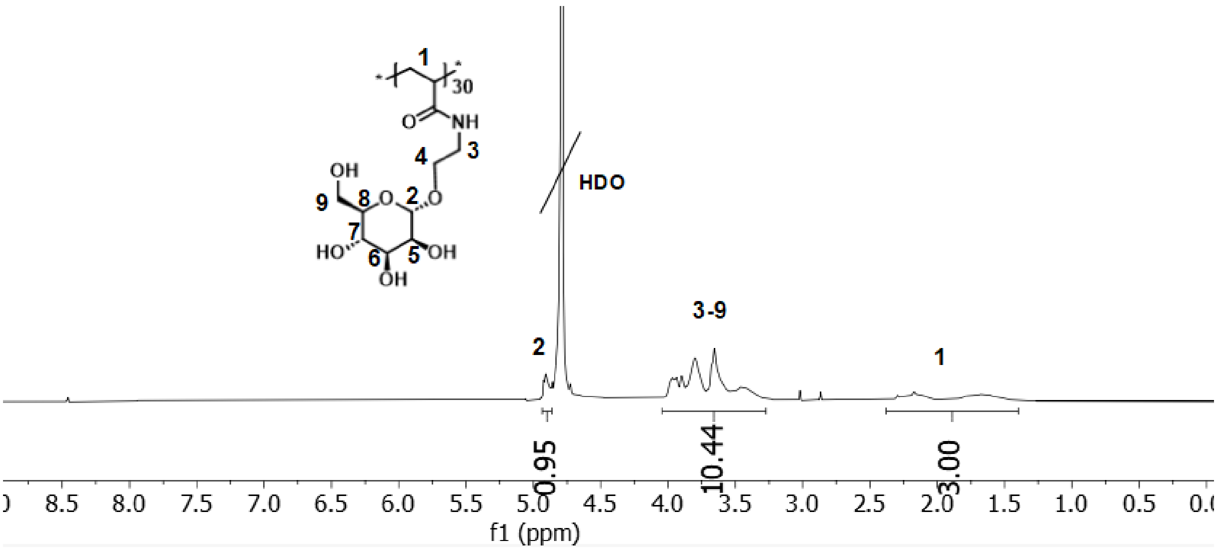

Figure S11. ^{1}H NMR spectrum (600 MHz, D_2O) of GP-30-OH: δ [ppm] 4.93-4.88 (m, **2,** D_2O overlapping), 4.04-3.25 (m, **3-9**), 2.37-1.38 (m, **1**).

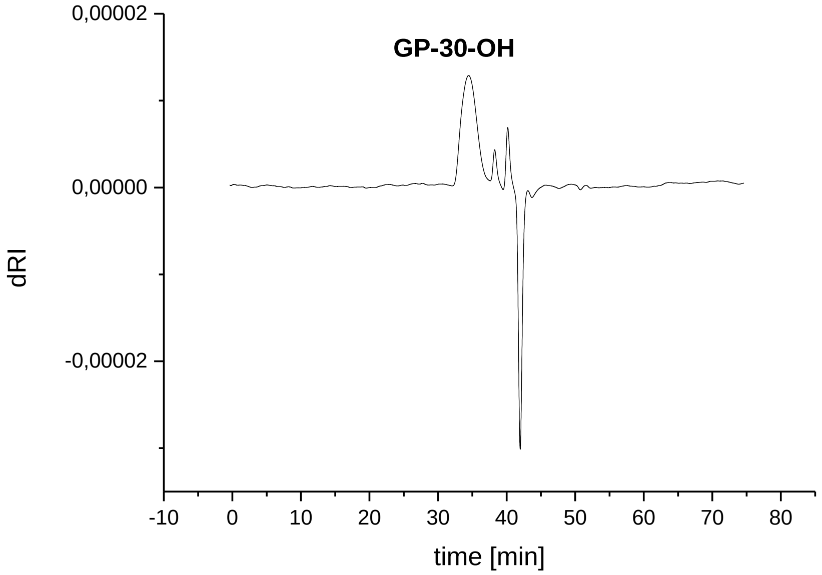

Figure S12. H_2O-SEC spectrum of GP-30-OH.

GP-70-OH (6)

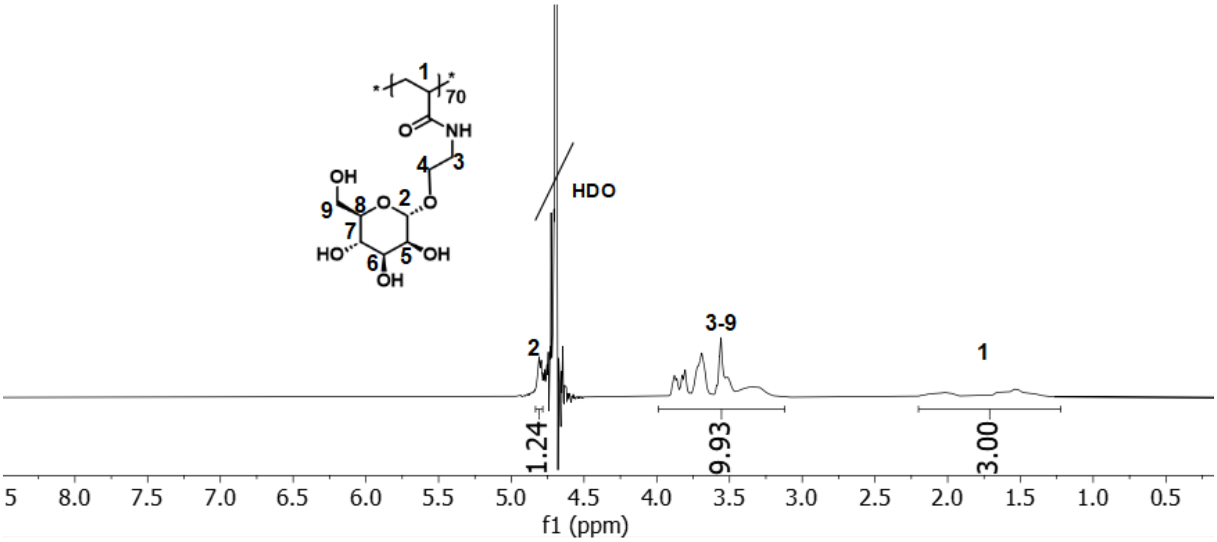

Figure S13. ^{1}H NMR spectrum (600 MHz, D_2O) of GP-70-OH: δ [ppm] 4.85-4.77 (m, **2,** D_2O overlapping), 3.98-3.11 (m, **3-9**), 2.19-1.22 (m, **1**).

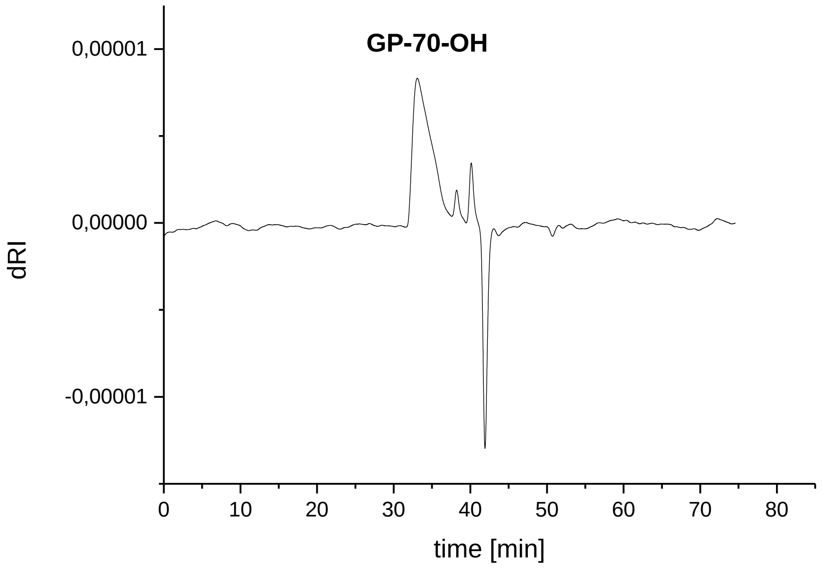

Figure S14. H_2O-SEC spectrum of GP-70-OH.

GP-200-OH (7)

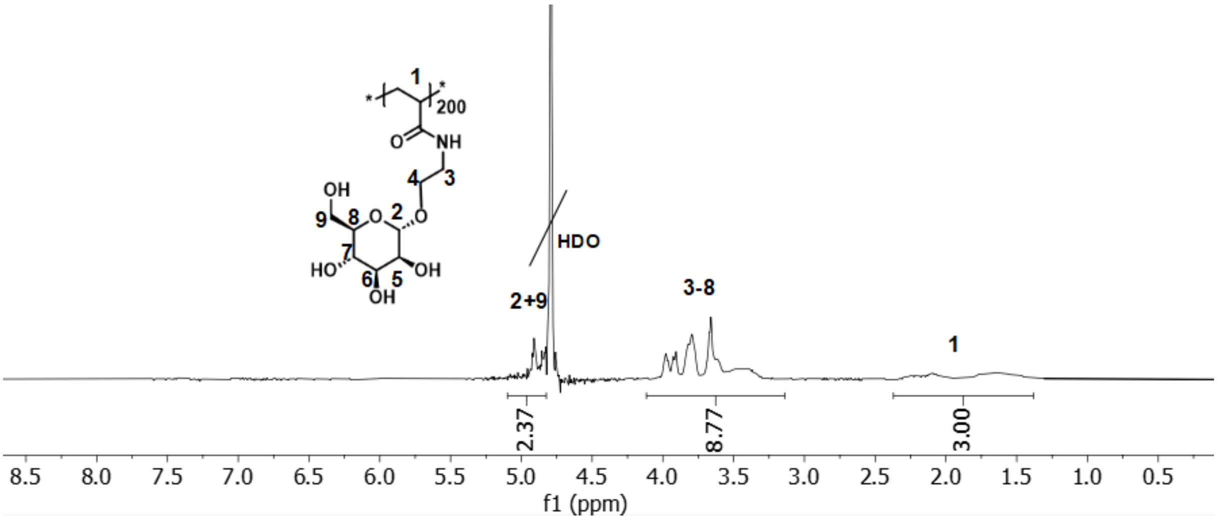

Figure S15. ^{1}H NMR spectrum (600 MHz, D_2O) of GP-200-OH: δ [ppm] 5.00-4.83 (m, **2+9,** D_2O overlapping), 4.1-3.21 (m, **3-8**), 2.35-1.38 (m, **1**).

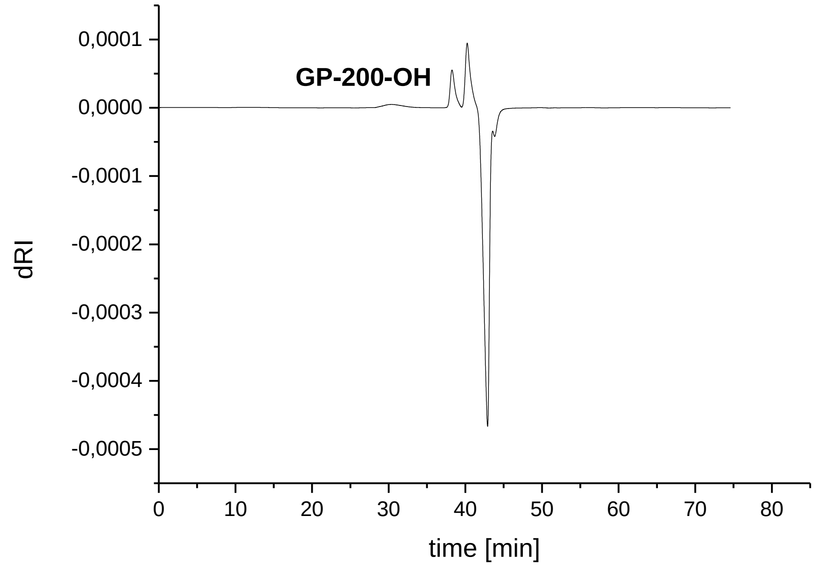

Figure S16. H_2O-SEC spectrum of GP-200-OH.

GP-300-OH (8)

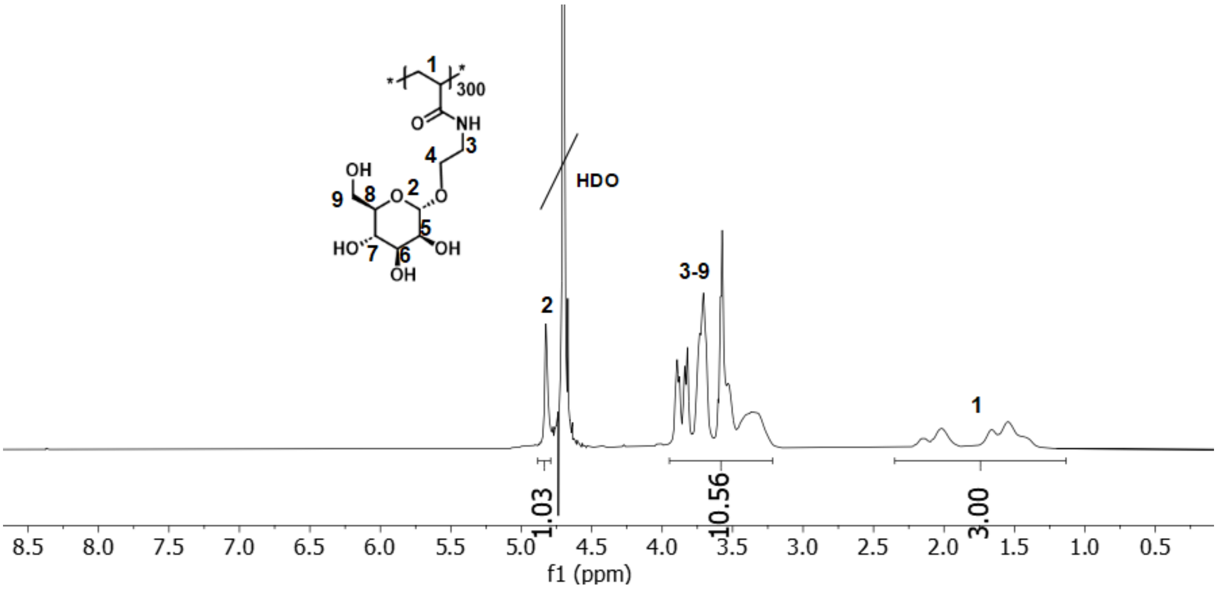

Figure S17. ^{1}H NMR spectrum (600 MHz, D_2O) of GP-300-OH: δ [ppm] 4.87-4.79 (m, **2,** D_2O overlapping), 3.95-3.2 (m, **3-9**), 2.3-1.19 (m, **1**).

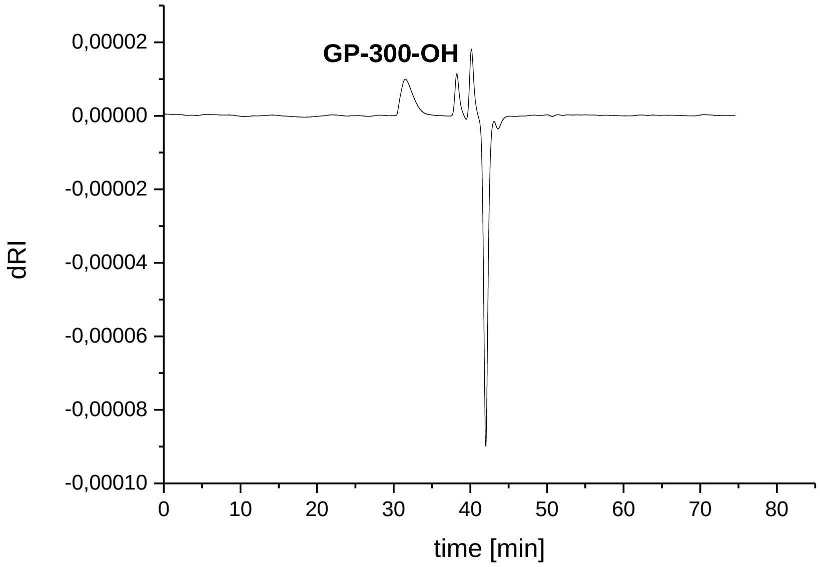

Figure S18. H_2O-SEC spectrum of GP-300-OH.

GP-800-OH (9)

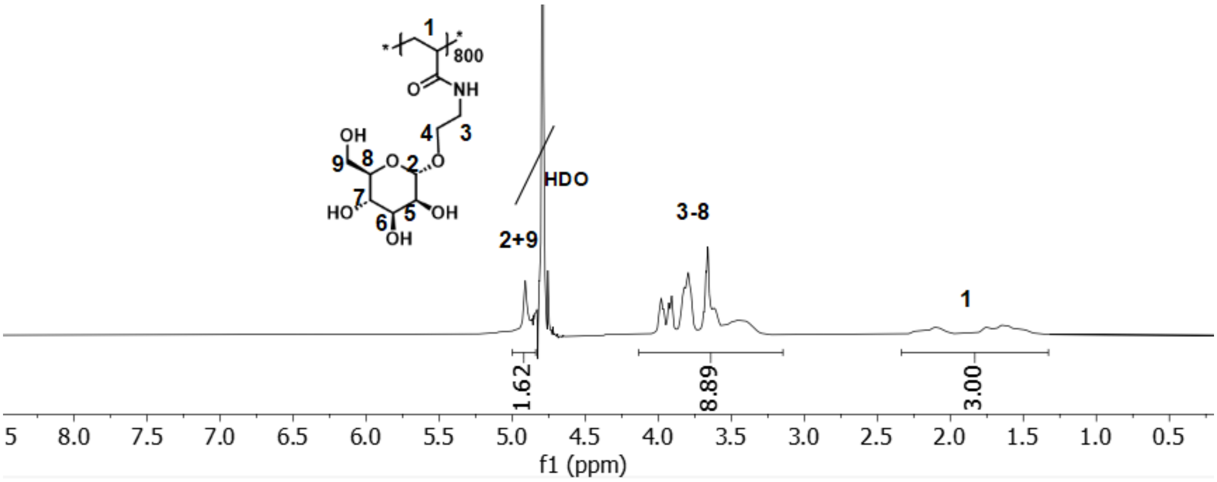

Figure S19. ^{1}H NMR spectrum (600 MHz, D_2O) of GP-800-OH: δ [ppm] 5.00-4.83 (m, **2+9,** D_2O overlapping), 4.07-3.24 (m, **3-8**), 2.3-1.32 (m, **1**).

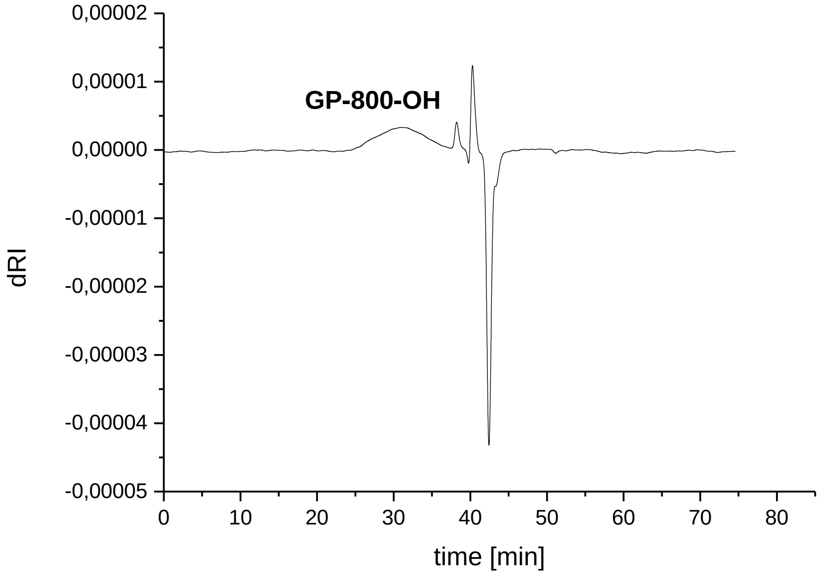

Figure S20. H_2O-SEC spectrum of GP-800-OH.

PHEAA-200-OH (10)

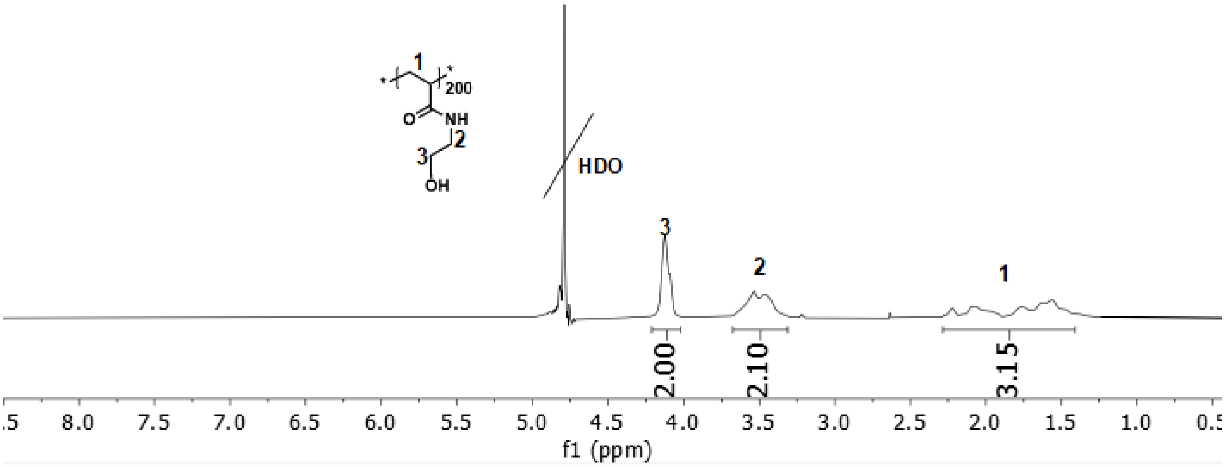

Figure S21. ^{1}H NMR spectrum (600 MHz, D_2O) of PHEAA-200-OH: δ [ppm] 4.18-4.02 (m, **3**), 3.64-3.30 (m, **2**), 2.25-1.36 (m, **1**).

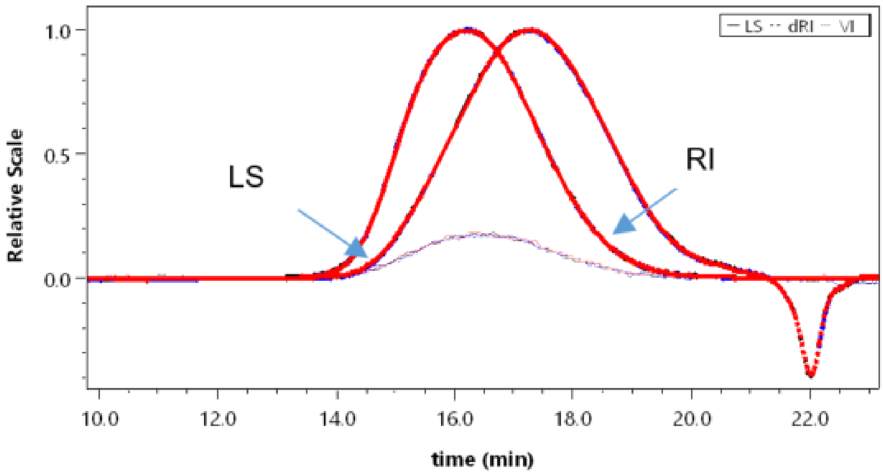

Figure S22. H_2O-SEC-MALS and H_2O-RI-SEC spectra (measured at Leibniz Institute of polymer research in Dresden) of PHEAA-200-OH.

coGP-70-OH (30%) (11)

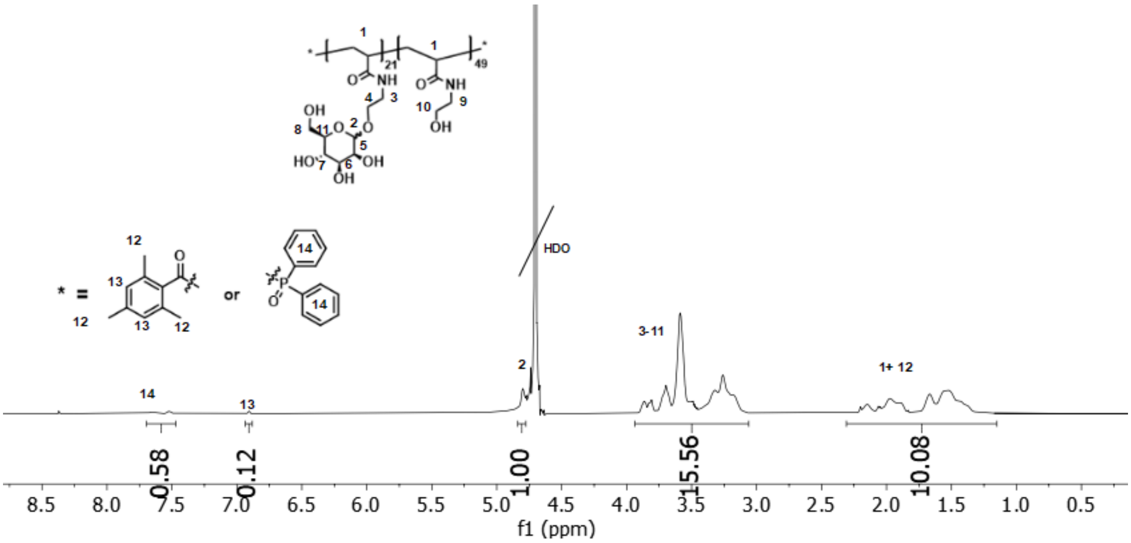

Figure S23. ^{1}H NMR spectrum (600 MHz, D_2O) of coGP-70 (30%)-OH: δ [ppm] 7.79-7.59 (m, **14**), 7.00-6.99 (m, **13**), 4.82-4.80 (m, **2,** D_2O overlapping), 3.93-3.04 (m, **3-11**), 2.20-1.17 (m, **1+12**).

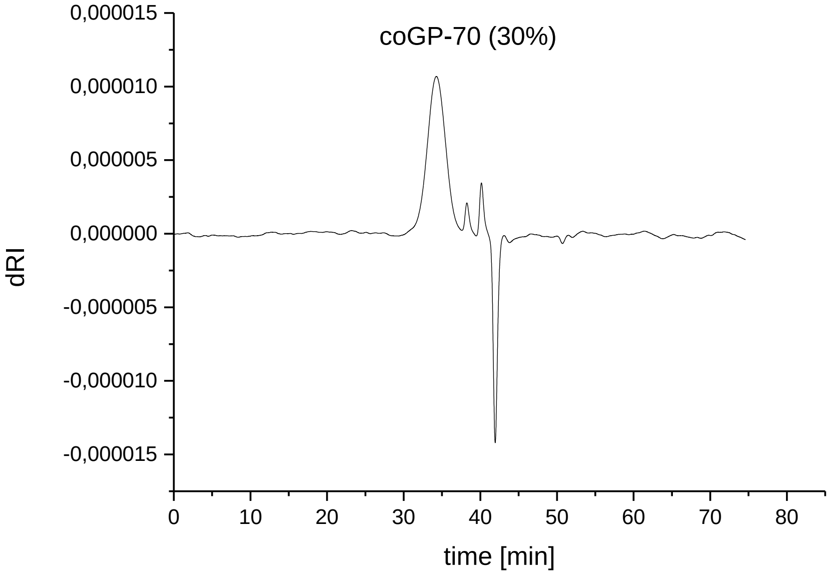

Figure S24. H_2O-SEC spectrum of coGP-70 (30%)-OH.

coGP-70-OH (50%) (12)

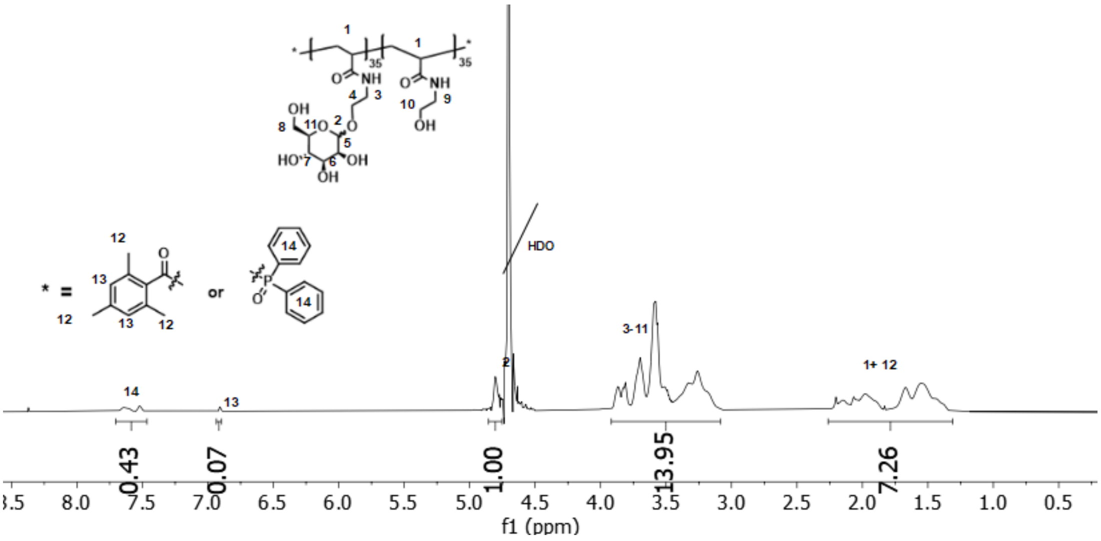

Figure S25. ^{1}H NMR spectrum (600 MHz, D_2O) of coGP-70 (50%)-OH: δ [ppm] 7.79-7.59 (m, **14**), 7.00-6.99 (m, **13**), 4.82-4.80 (m, **2,** D_2O overlapping), 3.93-3.04 (m, **3-11**), 2.20-1.17 (m, **1+12**).

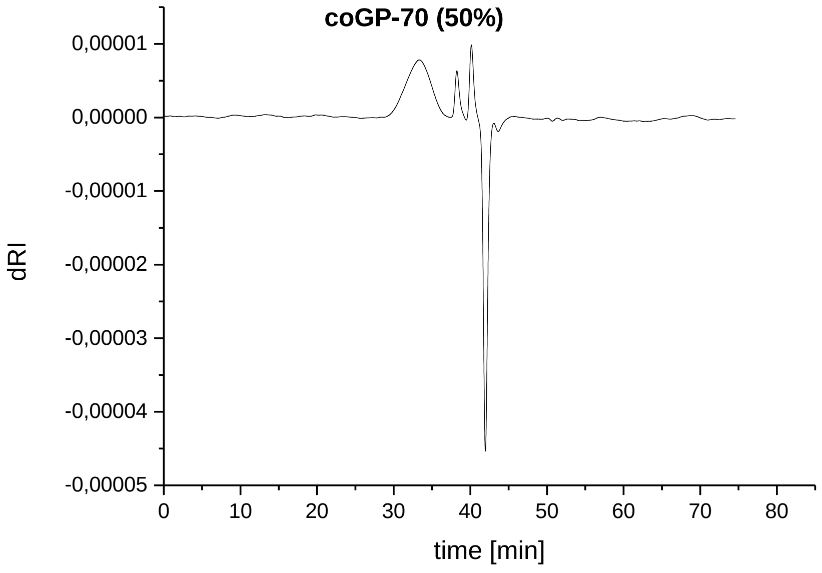

Figure S26. H_2O-SEC spectrum of coGP-70 (50%)-OH.

coGP-70-OH (70%) (13)

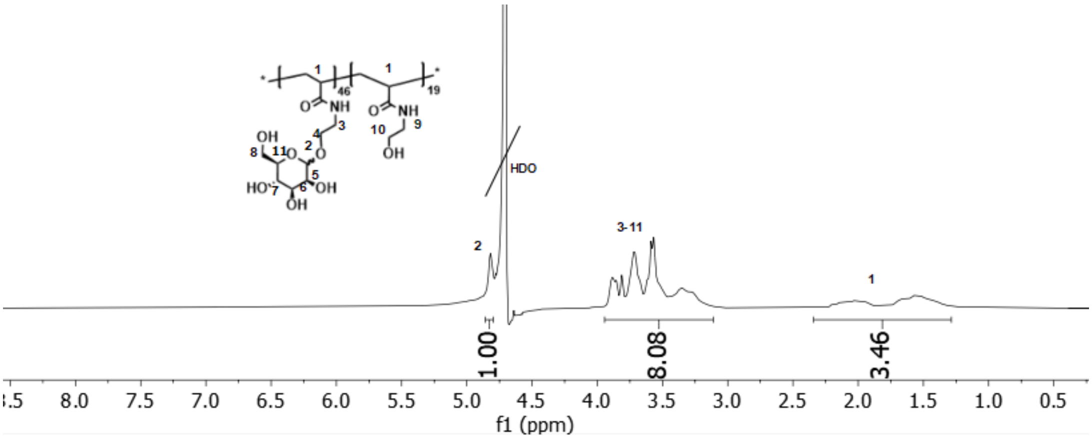

Figure S27. ^{1}H NMR spectrum (600 MHz, D_2O) of coGP-70 (70%)-OH: [ppm] 4.82-4.80 (m, **2,** D_2O overlapping), 3.95-3.13 (m, **3-11**), 2.34-1.29 (m, **1**).

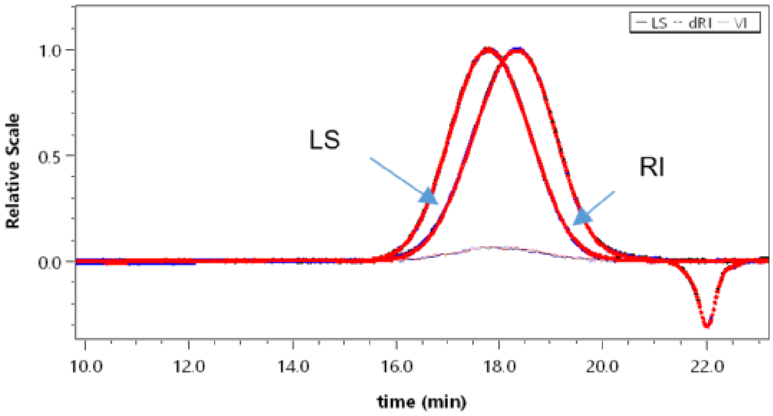

Figure 28. H_2O-SEC-MALS and H_2O-RI-SEC spectra (measured at Leibniz Insitute of polymer research in Dresden) of coGP-70 (70%)-OH.

coGP-300-OH (50%) (14)

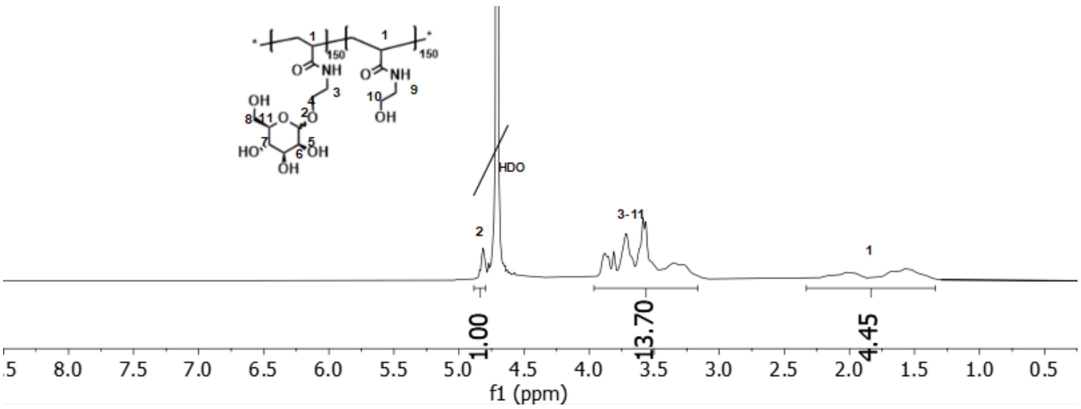

Figure S29. ^{1}H NMR spectrum (600 MHz, D_2O) of coGP-300 (50%)-OH: δ [ppm] 4.82-4.80 (m, **2,** D_2O overlapping), 3.97-3.10 (m, **3-11**), 2.34-1.31 (m, **1**).

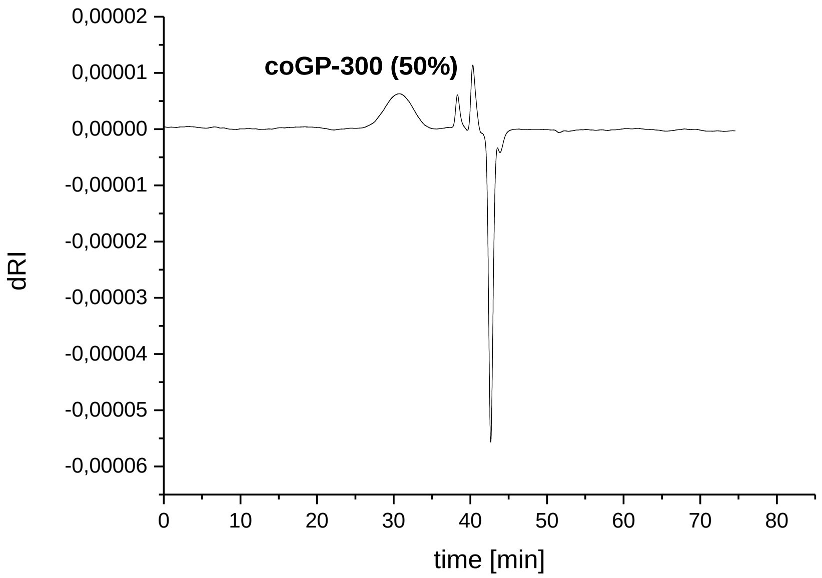

Figure S30. H_2O-SEC spectrum of coGP-300 (50%)-OH.

GP-70-nl (3S)

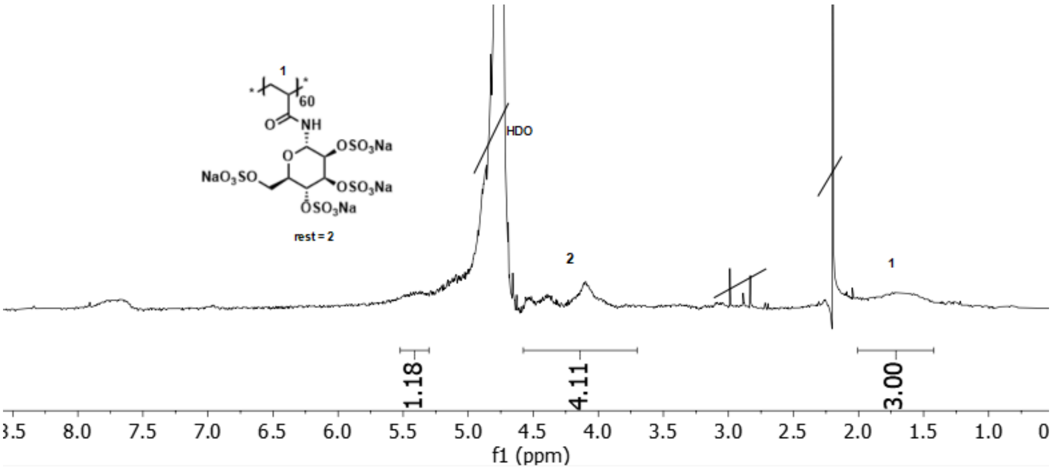

Figure S31. ^{1}H NMR spectrum (600 MHz, D_2O) of GP-70-nl: δ [ppm] 5.51-3.68 (m, **2**, D_2O overlapping), 1.98-1.42 (m, **1**).

GP-10 (4S)

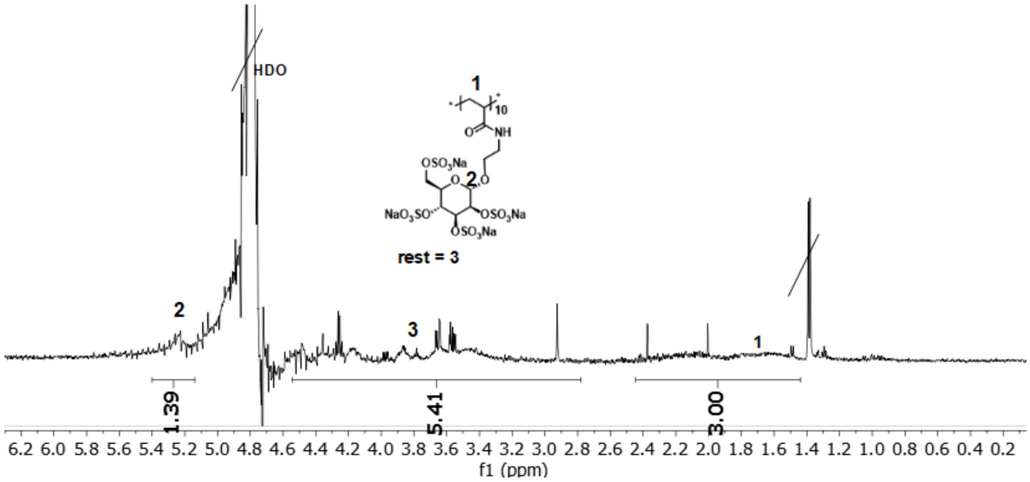

Figure S32. ^{1}H NMR spectrum (600 MHz, D_2O) of GP-10: δ [ppm] 5.38-5.13 (m, **2**), 4.49-2.80 (m, **3**, D_2O overlapping), 2.50-1.4 (m, **1**).

GP-30 (5S)

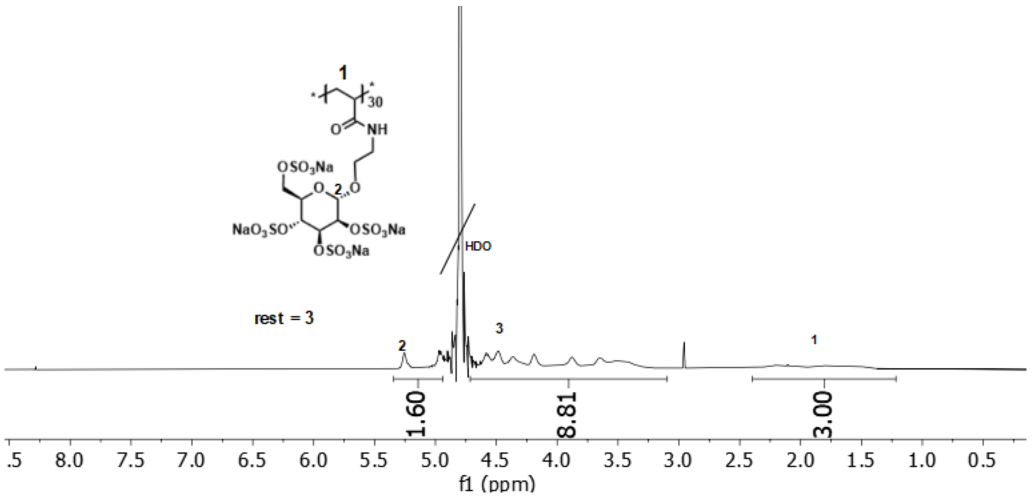

Figure S33. ^{1}H NMR spectrum (600 MHz, D_2O) of GP-30: δ [ppm] 5.29-5.18 (m, **2**), 5.01-3.23 (m, **3**, D_2O overlapping), 2.40-1.23 (m, **1**).

GP-70 (6S)

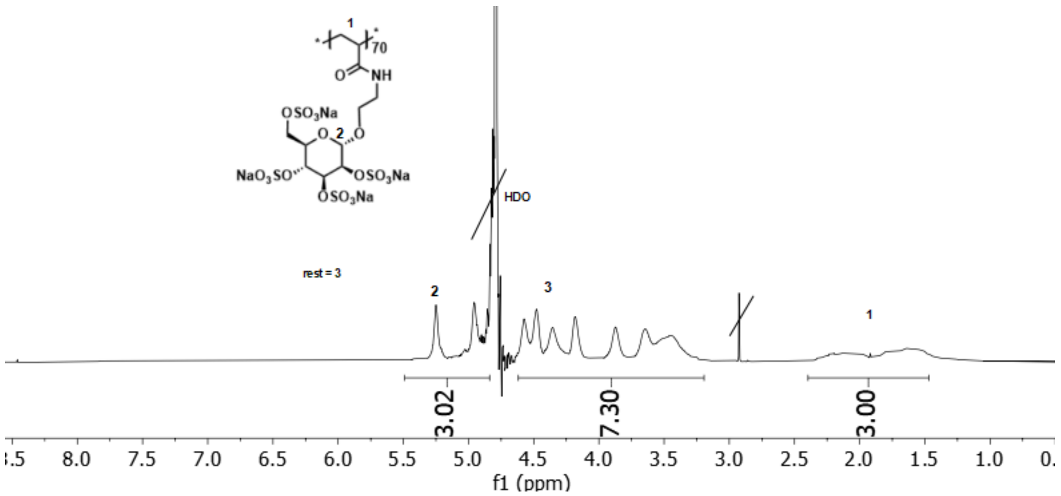

Figure S34. ^{1}H NMR spectrum (600 MHz, D_2O) of GP-70: δ [ppm] 5.30-5.18 (m, **2**), 5.09-3.22 (m, **3**, D_2O overlapping), 2.38-1.38 (m, **1**).

GP-200 (7S)

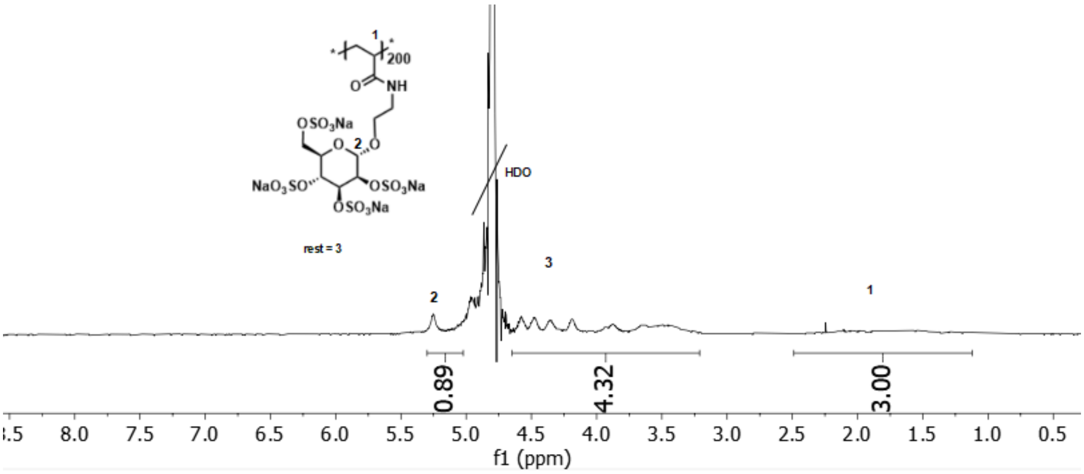

Figure S35. ^{1}H NMR spectrum (600 MHz, D_2O) of GP-200: δ [ppm] 5.30-5.18 (m, **2**), 5.05-3.19 (m, **3**, D_2O overlapping), 2.46-1.12 (m, **1**).

GP-300 (8S)

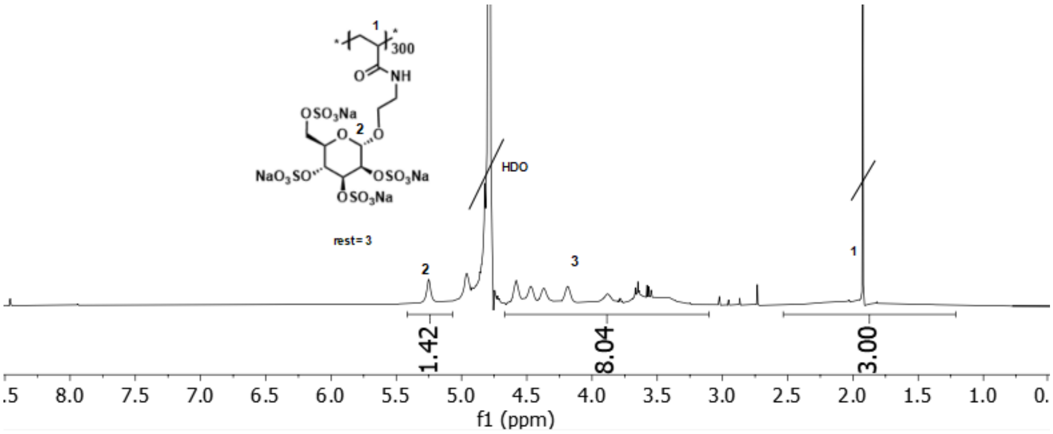

Figure S36. ^{1}H NMR spectrum (600 MHz, D_2O) of GP-300: δ [ppm] 5.32-5.21 (m, **2**), 5.04-3.14 (m, **3**, D_2O overlapping), 2.50-1.22 (m, **1**).

GP-800 (9S)

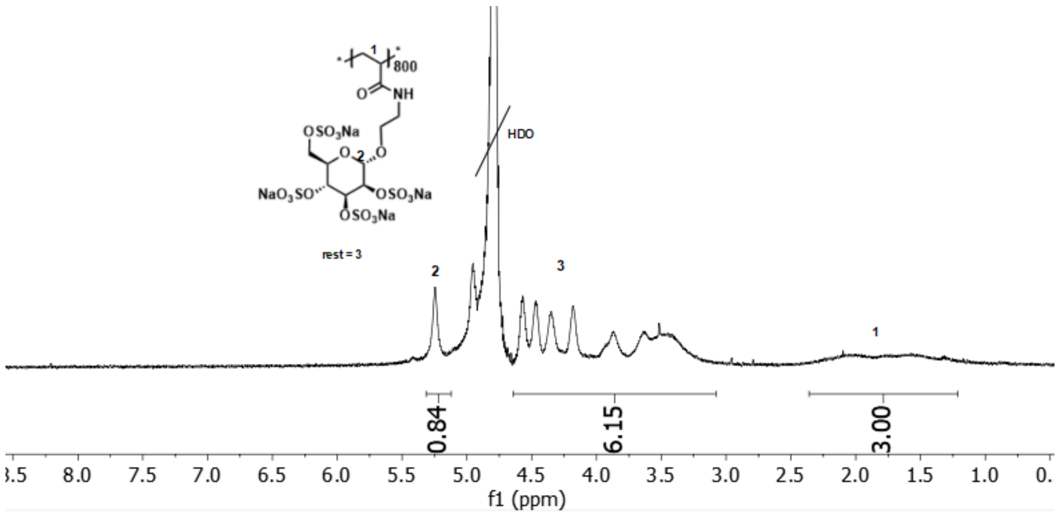

Figure S37. ^{1}H NMR spectrum (600 MHz, D_2O) of GP-800: δ [ppm] 5.30-5.18 (m, **2**), 5.02-3.11 (m, **3**, D_2O overlapping), 2.35-1.23 (m, **1**).

PHEAA-200 (10S)

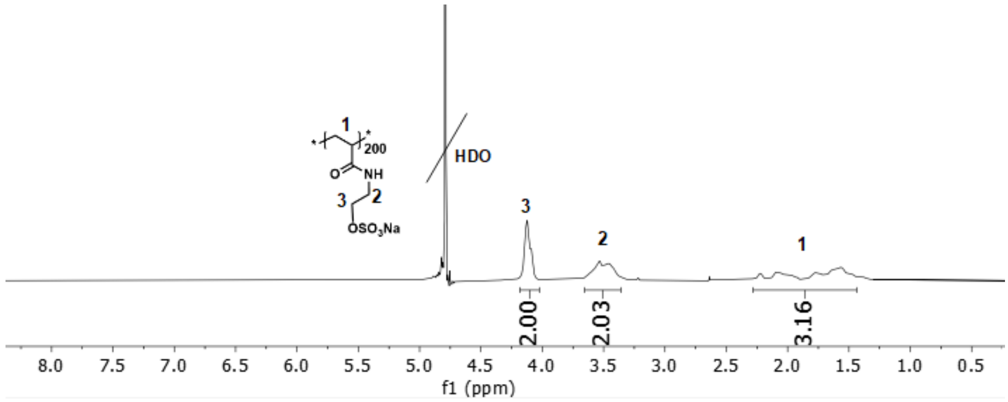

Figure S38. ^{1}H NMR spectrum (600 MHz, D_2O) of PHEAA-200: δ [ppm] 4.18-4.03 (m, **3**), 3.62-3.33 (m, **2**), 2.25-1.36 (m, **1**).

coGP-70 (30%) (11S)

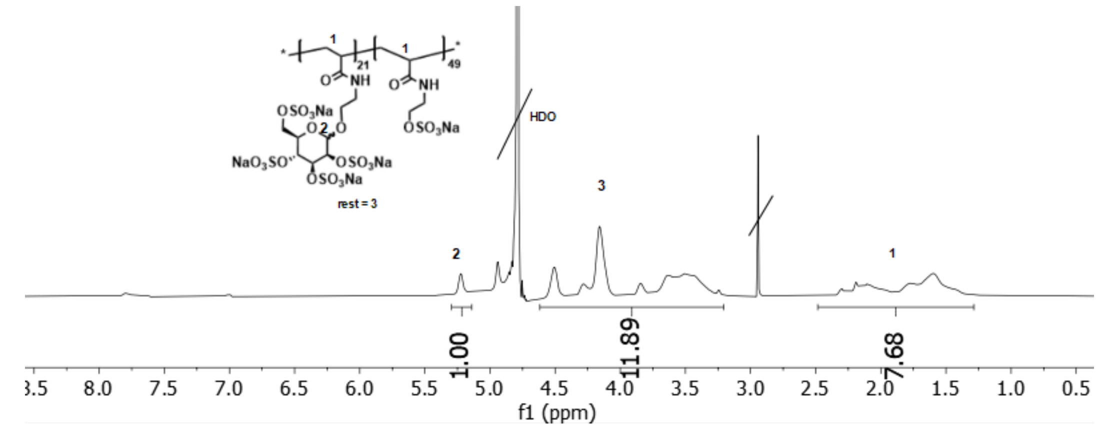

Figure S39. ^{1}H NMR spectrum (600 MHz, D_2O) of coGP-70 (30%): δ [ppm] 5.27-5.17 (m, **2**), 4.96-3.21 (m, **3**, D_2O overlapping), 2.42-1.31 (m, **1**).

coGP-70 (50%) (12S)

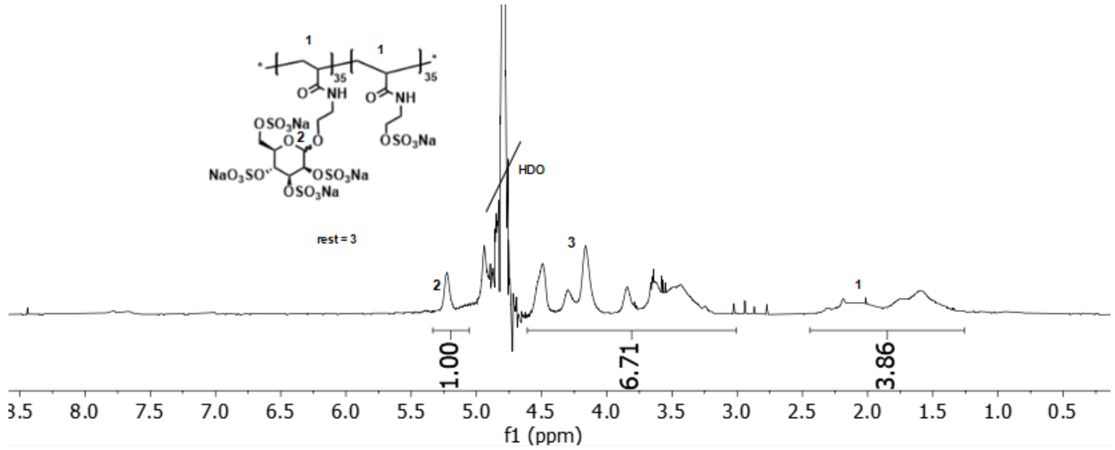

Figure S40. ^{1}H NMR spectrum (600 MHz, D_2O) of coGP-70 (50%): δ [ppm] 5.27-5.17 (m, **2**), 4.98-3.04 (m, **3**, D_2O overlapping), 2.42-1.24 (m, **1**).

coGP-70 (70%) (13S)

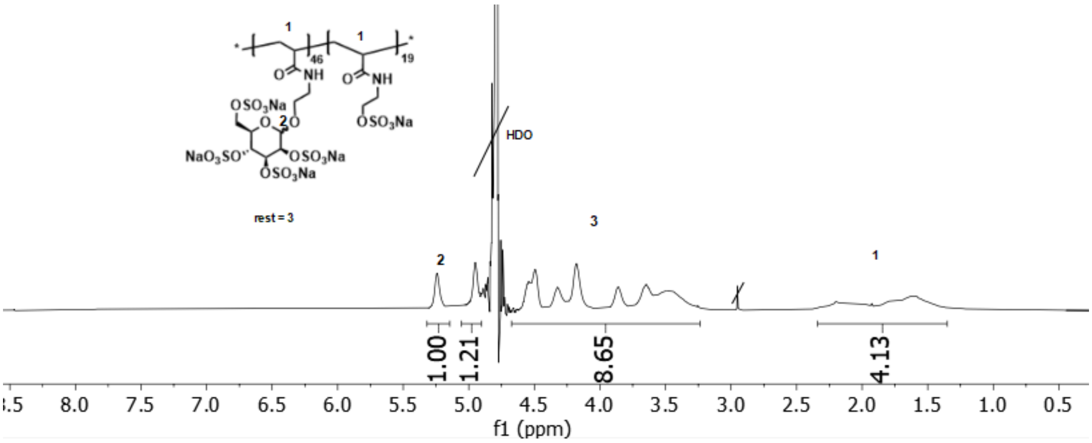

Figure S41. ^{1}H NMR spectrum (600 MHz, D_2O) of coGP-70 (70%): δ [ppm] 5.29-5.19 (m, **2**), 5.03-3.24 (m, **3**, D_2O overlapping), 2.33-1.35 (m, **1**).

coGP-300 (50%) (14S)

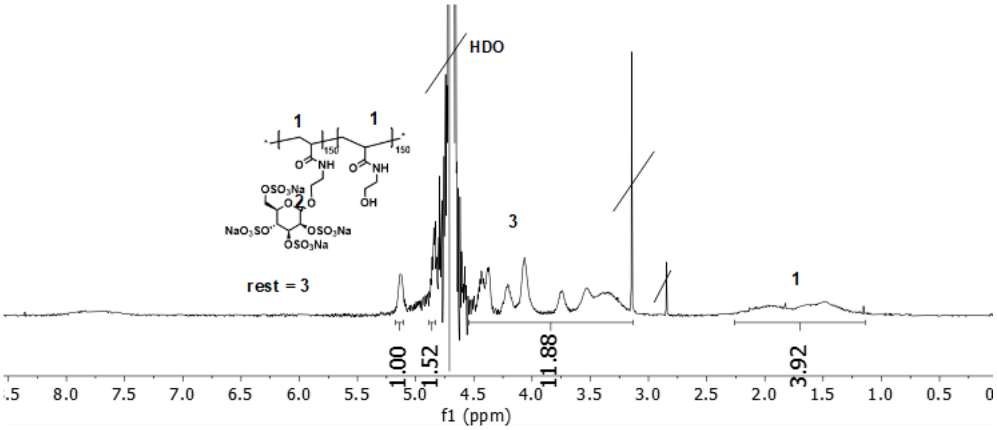

Figure S42. ^{1}H NMR spectrum (600 MHz, D_2O) of coGP-300 (50%): δ [ppm] 5.17-5.08 (m, **2**), 5.03-3.11 (m, **3**, D_2O overlapping), 2.24-1.12 (m, **1**).

Table S1. Dynamic light scattering data for sulfated glycopolymers. Each polymer was dissolved in PBS buffer (pH =7.4) with a concentration of 0.5 mg/mL. 0.8 mL each was filtered into a polystyrene cuvette and the hydrodynamic radius of the samples, as well as the PDI were determined via DLS measurement. Unsulfated GP-300-OH was measured as a control sample.

**Sample**	**Hydrodynamic Radius [nm]**	**PDI**
GP-10	5.74	0.211
	5.26	0.268
	5.24	0.258
GP-30	5.37	0.248
	5.79	0.272
	6.05	0.28
GP-70	10.17	0.233
	10.06	0.214
	10.58	0.233
GP-300-OH	13	0.141
	13.25	0.153
	13.77	0.187
GP-300	19.27	0.164
	18.8	0.139
	19.66	0.149
coGP-70 (30%)	4.98	0.227
	5.24	0.21
	5.91	0.255
coGP-70 (50%)	10.54	0.3
	10.08	0.298
	11.49	0.323
coGP-70 (70%)	10.04	0.473
	9.49	0.661
	9.55	0.76
coGP-300 (50%)	15.82	0.173
	16.88	0.255
	18.66	0.321

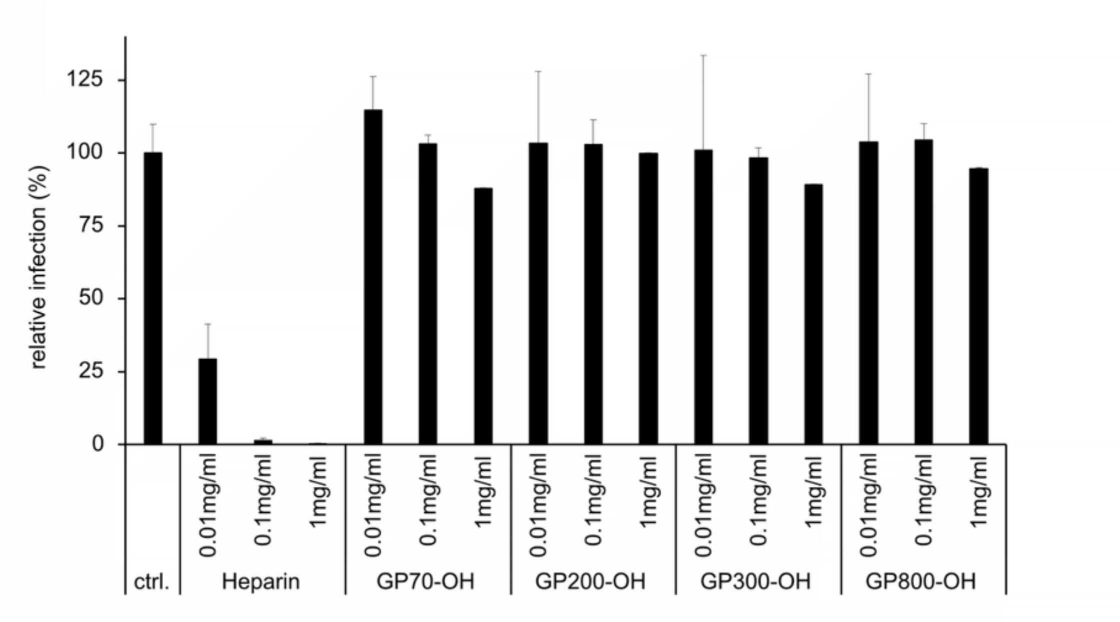

Figure S43. Inhibitory potential of glycomimetic compounds on SARS-CoV-2 infection. SARS-CoV-2 PsVs were incubated with glycosaminoglycans or glycomimetic polymers at the indicated concentrations for 1h. Subsequently, this mixture was added to cells for 1h, after which the inoculum was replaced by growth medium. Next, cells were fixed 24 h post infection (p.i.) and stained with RedDot for nucleus detection. The number of GFP-expressing (infected) cells was determined by automated microscopy and image analysis, normalized to the untreated control, and displayed as relative infection ± SD.
